# Coordinated inflammation and immune response transcriptional regulation in breast cancer molecular subtypes

**DOI:** 10.3389/fimmu.2024.1357726

**Published:** 2024-06-25

**Authors:** Tadeo Enrique Velazquez-Caldelas, Jose Maria Zamora-Fuentes, Enrique Hernandez-Lemus

**Affiliations:** ^1^ Computational Genomics Division, National Institute of Genomic Medicine, Mexico City, Mexico; ^2^ Center for Complexity Sciences, Universidad Nacional Autónoma de México, Mexico City, Mexico

**Keywords:** breast cancer, inflammation, immunity, network modularity, coexpression networks

## Abstract

Breast cancer, characterized by its complexity and diversity, presents significant challenges in understanding its underlying biology. In this study, we employed gene co-expression network analysis to investigate the gene composition and functional patterns in breast cancer subtypes and normal breast tissue. Our objective was to elucidate the detailed immunological features distinguishing these tumors at the transcriptional level and to explore their implications for diagnosis and treatment. The analysis identified nine distinct gene module clusters, each representing unique transcriptional signatures within breast cancer subtypes and normal tissue. Interestingly, while some clusters exhibited high similarity in gene composition between normal tissue and certain subtypes, others showed lower similarity and shared traits. These clusters provided insights into the immune responses within breast cancer subtypes, revealing diverse immunological functions, including innate and adaptive immune responses. Our findings contribute to a deeper understanding of the molecular mechanisms underlying breast cancer subtypes and highlight their unique characteristics. The immunological signatures identified in this study hold potential implications for diagnostic and therapeutic strategies. Additionally, the network-based approach introduced herein presents a valuable framework for understanding the complexities of other diseases and elucidating their underlying biology.

## 1 Introduction

The relationship between immune responses and breast cancer is a dynamic and multifaceted one, with profound implications for both tumor development and therapeutic strategies (1, 2). Breast cancer, one of the most prevalent malignancies worldwide, is characterized not only by the genetic alterations within cancer cells but also by the host’s immune system’s response to these abnormal cells (3–5). Immune responses play a pivotal role in shaping the tumor microenvironment, influencing disease progression, and determining treatment outcomes. While the immune system has the potential to recognize and eliminate cancer cells through surveillance mechanisms, breast tumors often employ sophisticated strategies to evade immune detection and suppression (6–8). Understanding the intricate connections between immune responses and breast cancer is vital for unraveling the complexities of this disease and developing innovative immunotherapeutic approaches that hold promise in improving patient outcomes.

Breast cancer tumors are composed of numerous interacting cell types. Among those cells, immune cells are often present to a varying degree in all tissues and participate in the many physiological and histological changes produced during inflammation. The role of the immune system in the development of tumors is not thoroughly understood. In some cases, the inflammatory microenvironment appears to provide the conditions for tumor growth and immune cell infiltrates are recognizable, whereas an active immune response is necessary to eliminate cancerous cells and limit tumor growth (9–12). Breast cancers present inherent variability, which impacts their behavior, evolution, and response to therapeutic interventions. Among the most widely used criteria in the decision-making process for treatment of breast cancer patients, tumors are sampled and classified based on a series of histological tests for the presence of molecular markers including steroid (progesterone and estrogen) hormone receptors, Her2 receptor, and the KI67 proliferation score, all of which are based on the identification of expressed proteins. Other classification schemes rely on the quantification of mRNA expression of panels of multiple genes such as the PAM50 classifier (13–15).

There is significant overlap between alternative tumor classification strategies that may be rooted on the underlying tumor biology. Classification reflects the similarities in behavior, gene expression, and most likely gene regulation. Gene regulation is the result of cell’s integration of internal (i.e., metabolic state and cell cycle state progression) and environmental signals (i.e., hormones, cytokines, and membrane contact receptors) according to their regulatory programs. Co-expression patterns are thus the resulting output of signaling events inside the cell (16, 17). The regulation of gene expression is a way in which the cell controls the availability of components of the cellular machinery that interact to assemble the mechanisms that drive cellular processes (18–20).

Here, we explore the co-regulation landscape of genes associated to the immune response in breast cancer molecular subtypes. By inspecting gene co-expression groups, we may get a hint of which mechanisms are likely to be used by each phenotype and possibly discern any major differences between the phenotypes that can help us understand their biological differences. We are doing this with an emphasis distinct from a single gene approach and with the assumption that we are looking at a tissue microenvironment level. We modeled the gene co-expression landscape as a network of interconnected genes. The network is a large-scale view of the phenomenon that allows us to review the global context of gene co-expression (21–23).

The intricate interplay between inflammation and immune responses in the context of breast cancer has emerged as a critical avenue of research, with the potential to shed light on the underlying molecular mechanisms driving this complex disease. In this study, we delve into the gene expression profiles within distinct molecular subtypes of breast cancer to elucidate the subtle yet pivotal variations in the coordination of inflammation and immune responses. By dissecting these intricate interactions at the gene expression level, we aim to uncover novel insights that may pave the way for more tailored therapeutic strategies and a deeper understanding of the disease’s heterogeneity. This investigation aims to contribute towards refining our comprehension of breast cancer biology and ultimately enhancing patient care.

## 2 Materials and methods

### 2.1 Data acquisition

Our dataset comes from a collection of Illumina HiSeq RNAseq datasets, consisting of 780 breast cancer tumor samples and 101 normal breast tissue samples obtained from The Cancer Genome Atlas (TCGA) project database Tomczak et al. (24). Datasets were downloaded and processed as described in Espinal-Enriquez et al. (25). The dataset contains information for a total of 15,267 expressed genes. Corresponding tumor samples were further classified into one of the four breast cancer intrinsic molecular subtypes: Luminal type A, Luminal type B, Her2-enriched, or Basal-like (referred to here as LumA, LumB, Her2, and Basal). In order to be able to dissect subtype specificities that may be veiled by marginally subtyped samples, we used a PAM50 classifier that is more stringent than the standard one (26). The rationale behind this is that the PAM50 classifier is employed to categorize patients into the breast cancer subtype with the highest correlation, regardless of the specific value obtained. However, to construct the risk of recurrence (ROR) score, which plays a crucial role in therapeutic decision-making, correlations with all subtypes are essential. Current estimations of subtype uncertainty lack accuracy, are infrequently taken into account, or demand a population-based approach within this context. After sample classification, tumor and normal mammary tissue samples were rejoined and batch effect was corrected as described in García-Cortés et al. (22). After classification, 415 samples were reliably assigned using the pbcmc R package (https://rdrr.io/bioc/pbcmc/) (26) to one of the breast cancer intrinsic molecular subtypes (**Table 1**).

**Table 1:** Number of samples corresponding to each breast cancer molecular intrinsic subtype.

Classification	Number of samples
Normal breast	101
Luminal A	143
Luminal B	58
Her2	72
Basal	142

After classification, 415 tumor samples were reliably assigned to one of the subtypes. communities can offer valuable insights into the potential co-occurrence of proteins within cells, hinting at possible interactions and contributions to biological processes.

### 2.2 Network construction

A network is a mathematical object that can be used to describe the interactions (referred as edges) within a collection of objects (nodes). In the context of gene expression, genes can be associated by the degree to which their expression levels are statistically dependent on the expression levels of other genes. If given a set of criteria, the expression levels between two genes are determined to be statistically dependent; this can be represented as an edge or interaction and included in the network’s structure (22, 23). Consequently, the network serves as a representation of the co-expression patterns found within the tissue from which the data originate. One notable structural feature is the presence of highly interconnected groups of co-expressed genes or communities. In these groups, if two genes are linked to a third gene within the same community, it is likely that they are also connected to each other. At the same time, communities tend to have sparser connections with the rest of the network. These communities can offer valuable insights into the potential co-occurrence of proteins within cells, hinting at possible interactions and contributions to biological processes.

To obtain the networks, we divided samples of each phenotype into five expression matrices, one for each of the four breast cancer subtypes and the healthy adjacent tissue. We used Shannon’s Mutual Information (MI) as a measure of statistical dependency of the expression level between pairs of genes. MI is a general, model-independent and non-parametric measure that can be reliably estimated from the empirical probability distributions of experimental data given large enough sample sizes (27). We obtained the MI values of all possible gene pairs from the 15,267 genes in the expression matrix, which means a total of 116,533,011 potential links (edges) between genes. MI values were calculated with the methodology implemented in the ARACNe algorithm (28), although DPI was not applied. The use of DPI was implemented in the ARACNe approach, in order to provide a way to disambiguate direct from indirect co-expression relationships in the context of a network inferred from two different gene sets, one of Transcription Factor genes and another one with the whole transcriptome (a so-called Regulon set network). This is so since it was aimed to provide not just co-expression networks but indeed an (undirected) approximation to gene regulatory networks. In the present case, gene co-expression networks are analyzed in the search of patterns leading to functional signatures: i.e., genes are co-expressed driven by a functional constraint or *necessity*, e.g., to activate/repress biological processes. In this regard, under this guilt-by-association approach, the distinction between direct and indirect associations is not that relevant. For those reasons, we did not use the data processing inequality (Markov information decay) in our networks. Gene pair interactions were sorted by descending MI value and a cutoff was established at the top 100,000 valued pairs, which corresponds to the top 0.08% links (22, 25).

Also relevant to mention is how MI thresholding (i.e., establishing a minimum MI value to consider that a statistical dependency relationship is significant, also called network pruning) was carried out. Full transcriptome statistical dependency matrices in humans have approximately 200 million independent entries (these are symmetric matrices). By means of statistical analyses, heuristic bootstrap studies, and network topology calculations, our research group has observed in the past (supported by extensive bootstrapping calculations, simulations, and statistical tests) (25, 29) that, depending on the specificities of the background noise of the underlying experiments and the intrinsic variability of the samples, between 0.01% and 0.1% of these dependencies are strong enough to represent biologically relevant co-expression relations in cancer, thus forming the basis for co-expression networks. That was the rationale used here to retain the top 100,000 higher MI links in the different networks.

### 2.3 Community identification and statistical overrepresentation analysis

Modularity analysis was performed in the co-expression networks (30). The Infomap algorithm was used to this end (31, 32). For each phenotype, we obtained a sub-network from the top 100,000 MI interaction values. Each sub-network consists of many independent (there is no link path between them) groups of genes, called components, which vary in the number of associated genes. Within each component of the network, genes (the nodes in the network) are not uniformly connected. We can distinguish groups of genes that are densely interconnected, but less connected to the rest of the network. These groups are called communities (for a comparative view of the different networks and their modules see **Table 2**). To assess the community structure in our networks, we used the community detection algorithm infomap (31). We further considered communities with at least 10 genes as likely representative of possible biological functions. We labeled communities by combining the (automatically assigned) number of the community in the network and the phenotype to which this network corresponds. To give us an idea of the possible biological functions that may be regulated at the gene expression level with a relatively fine level of granularity, the gene lists of each valid community were tested with a statistical overrepresentation analysis (ORA) for the categories of the Biological Process Ontology defined by the Gene Ontology consortium (33). Enrichment values were assessed for statistical significance by means of hypergeometric permutation tests with FDR-corrected *p*-values less than 0.05. In what follows, we will use the term *enriched communities* as a short-hand for Community (or module) with statistically significant categories on a gene set enrichment analysis.

**Table 2:** General comparison between the top 100k interactions networks.

Metric	Normal breast	LumA	LumB	Her2	Basal
Number of genes	12,930	12,844	12,599	13,739	12,301
Modularity	0.47	0.88	0.89	0.84	0.89
Total communities	976	956	956	993	944
Communities with 10 or more genes	321	187	170	222	176
Number of enriched communities	48	48	39	49	44
Communities with immune system enrichments	11	14	11	12	13

### 2.4 Community comparisons

We compared communities at three complementary levels. First, we looked at functional enrichment to see functional patterns within the same network and between networks. Second, we compared the gene composition of the communities between networks to see if the similarities at the functional level were a reflection of similar gene composition. Finally, we compared the structure of the communities looking at the gene-to-gene associations (also called interactions or edges). Community similarity was determined using the Jaccard similarity index, which is the size of their intersection divided by the size of their union.

### 2.5 Differential expression analysis

Differential expression analysis was performed with the Limma package (34) from Bioconductor Ver. 3.8.0, using R Ver. 3.5.1. The statistical method used was empirical Bayes and the criteria for differential expression was −1≤log fold change ≥1 and *B* statistic ≥6.

## 3 Results

As our group have previously observed, the networks inferred from each one of the subtypes and the normal breast tissue show differences in gene composition and have distinct overall structure, although our dataset considers the same gene lists as a starting point (35). These differences arise from subtle distinctions in the association of genes into co-expression groups as well at the internal structure of groups that share a portion of genes (22, 25).

**Figure 1:**
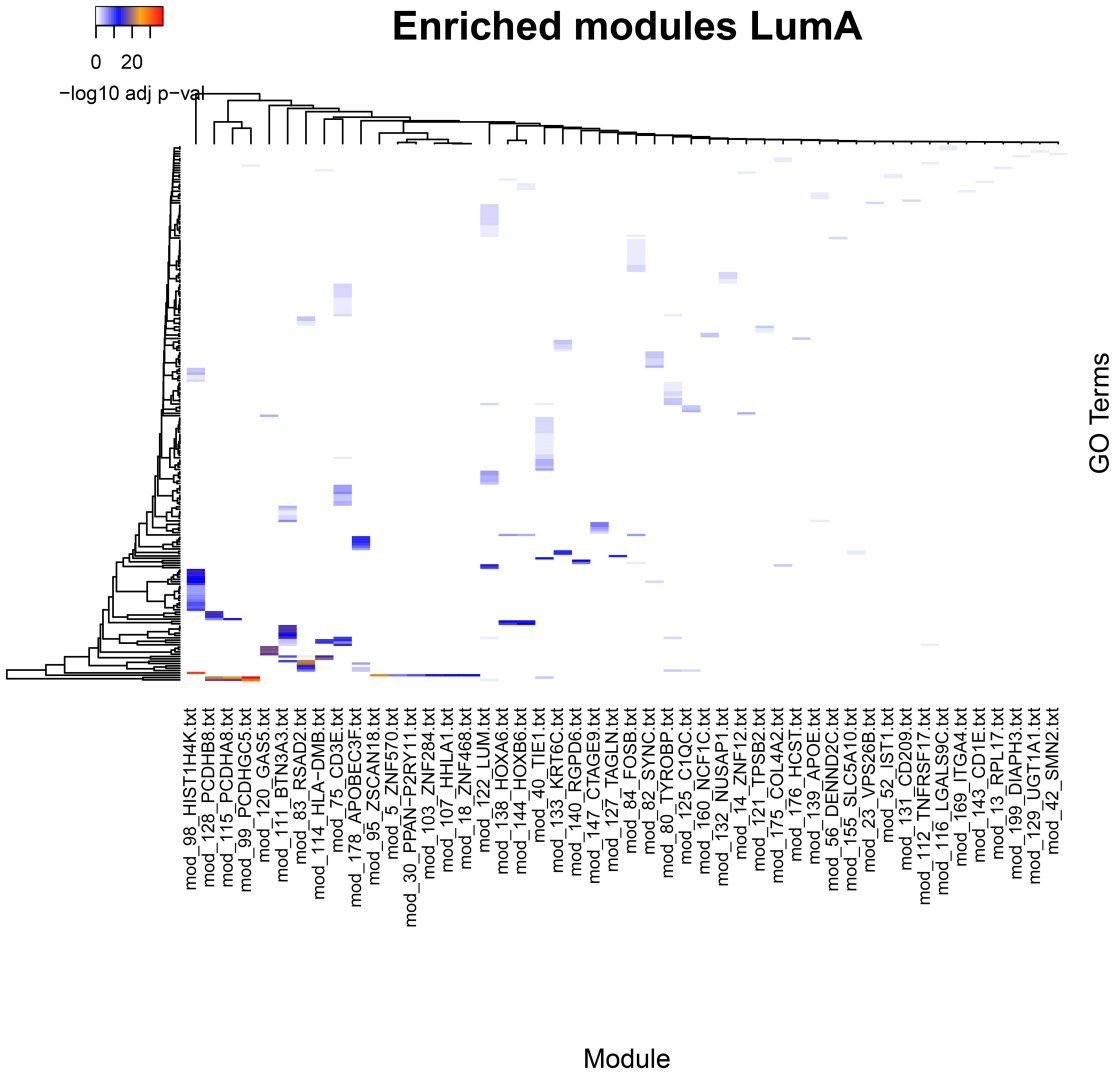
Significantly enriched categories in GO Biological Process for the communities in the Luminal A breast cancer subtype network. Although only one breast cancer subtype is presented, it is representative of the pattern of enrichment classes in which each community presents characteristic, non-shared enrichment categories, which suggests functional specialization of the communities (see **Supplementary Figures 1**–**3**). Processes that are shared by multiple modules are few and defined in a very general manner. A line spanning one of the lower rows through many communities labeled with genes for the ZNF family of proteins. Such communities contain multiple genes annotated as transcription factors and contribute to the significance in the enrichment of the rather general process GO:0006357 Regulation of transcription by RNA polymerase II.

**Figure 2:**
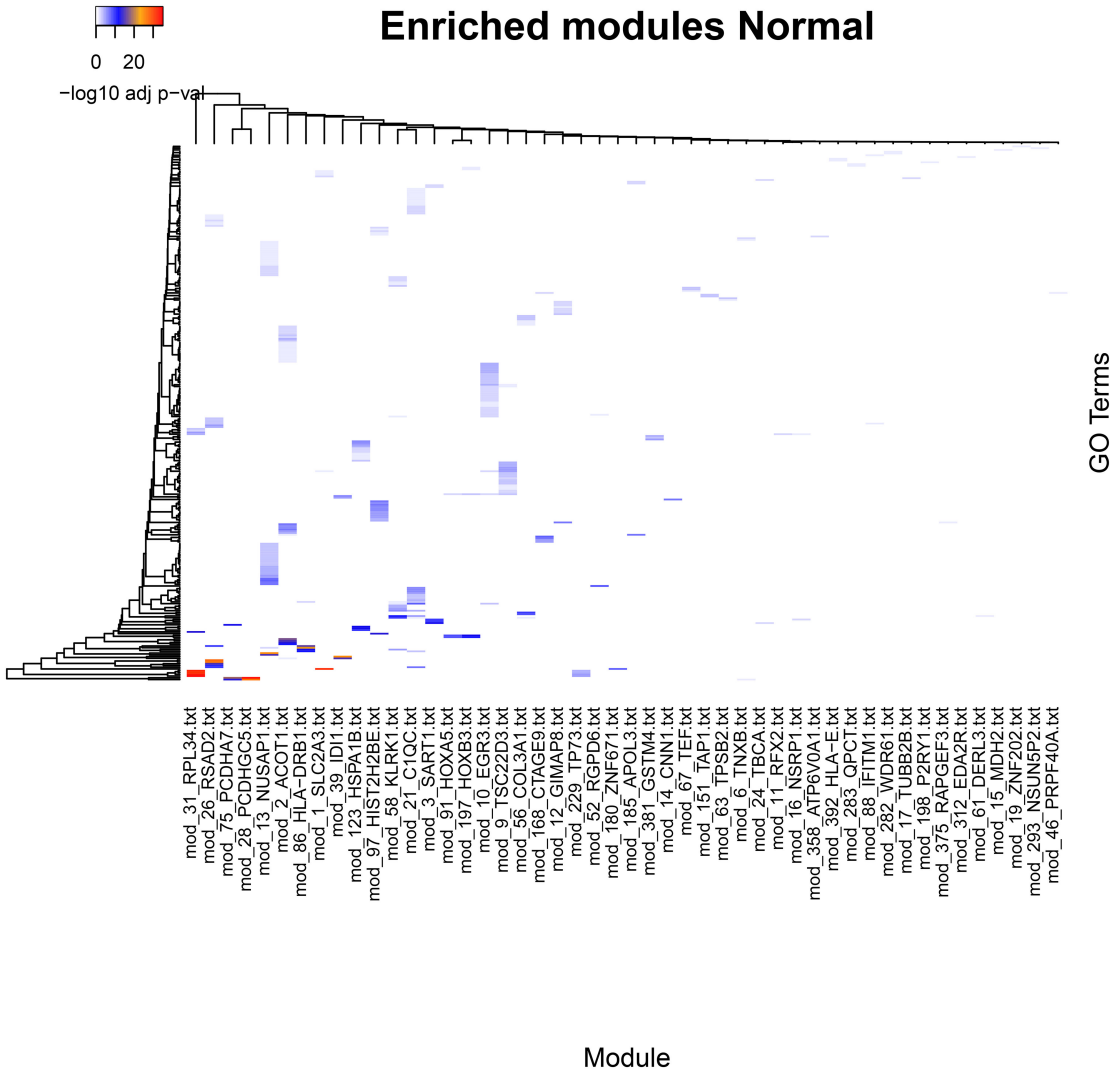
Significantly enriched categories in GO Biological Process for the communities in the Normal (healthy) tissue network. Statistically significant categories differ from the Cancer networks (see Supplementary data for further details).

The comparison of community enrichment profiles within a network shows distinct biological processes characteristic of each community. This is consistent with our previous observations from breast cancer expression datasets (36). The number of enriched processes in each community varies from one to a few tens, although the modules contain only a few genes for each category. The functional separation of the communities is made apparent when we visually compare the enriched categories in each network (**Figures 1**, **2**).

An overview of the enriched categories on each network provides a general idea of the complexity of underlying functions driving a phenotype. Each one of the enrichment sets includes a wide array of processes.

Processes enriched in the normal phenotype are biased towards cellular metabolism, energy production, lipid biosynthesis, tissue structure, and immune surveillance and response. In general, these functions are congruent with tissue maintenance and metabolism. In contrast, tumor phenotypes show a number of processes associated to an active immune response, active tissue remodeling, morphogenesis, angiogenesis, and wound healing. We found also a number of processes associated to immune cell migration, activation, proliferation, and effector function. This pattern is shared although with differences between each tumor subtype.

In all phenotypes, a recurring theme is the presence of multiple instances of regulation of gene expression where multiple communities show one or more GO terms associated with it. These communities contain genes for transcription factors. An interesting difference in tumor phenotypes is the presence to a higher degree of multiple process enrichments for mRNA processing, mRNA expression regulation by miRNAs, translation regulation, and post-translational regulation by protein degradation.

**Figure 3:**
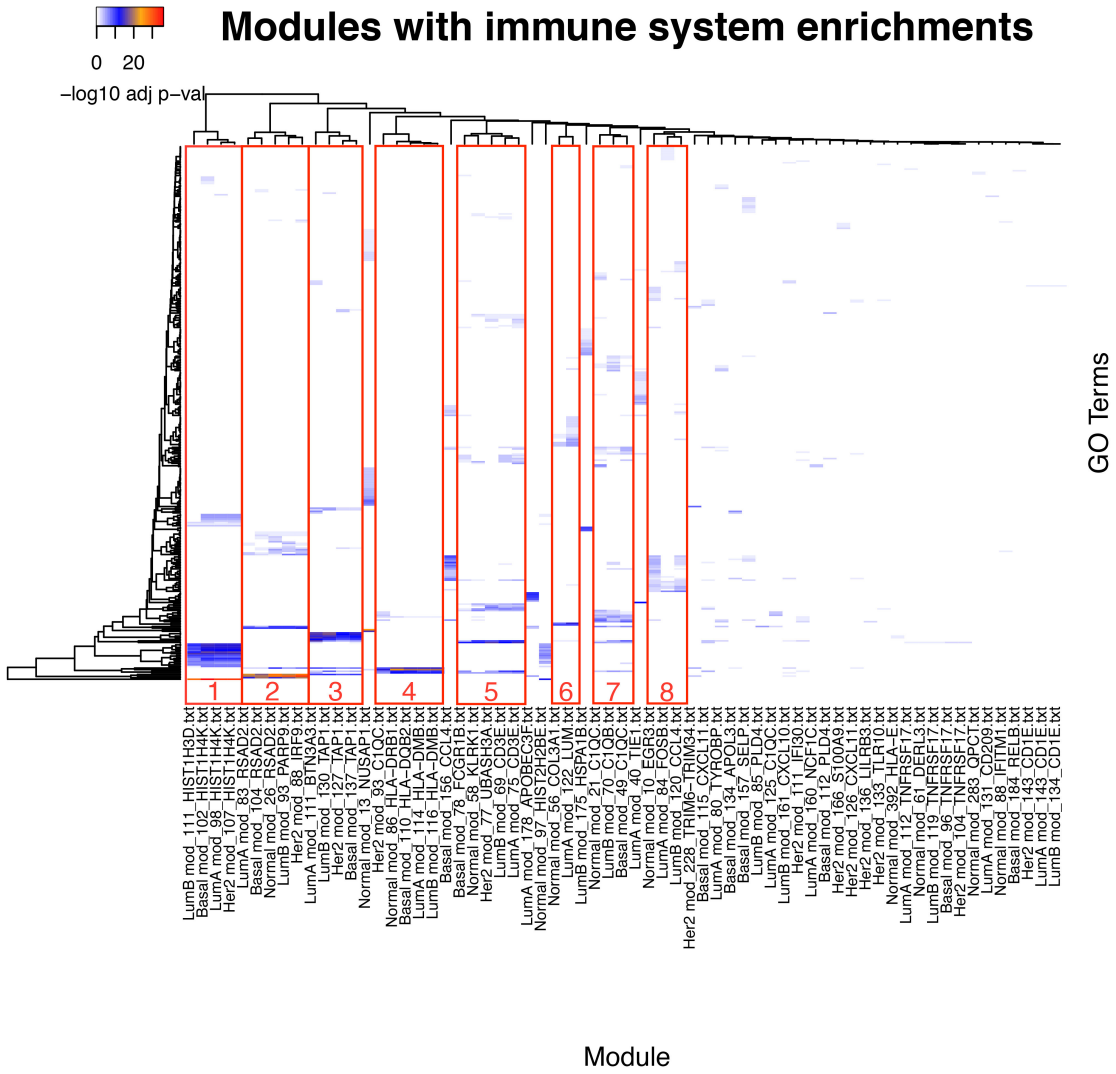
Comparison of the communities with inflammation and immune system enrichment categories from all networks. Clusters of communities with similar enrichment patterns can be observed; some of the enriched categories appear in all networks.

**Figure 4:**
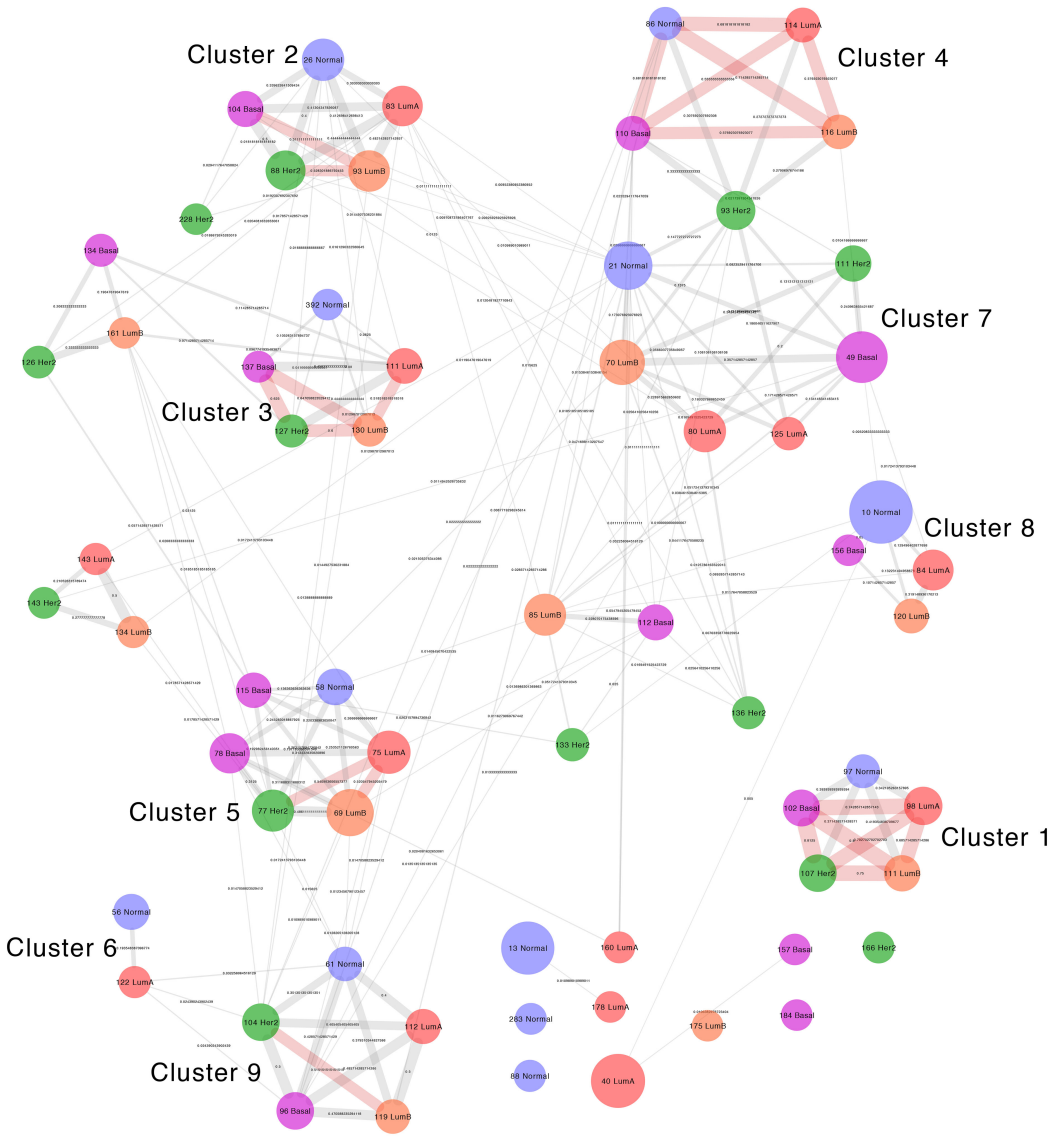
Visualization of the Jaccard Similarity index between communities with immune system enrichment categories from all networks. Groups of communities with high similarity can be observed and such groups correspond to the clusters in the left panel. Nodes represent communities, which are color coded and named based on the network of origin. Node size is proportional to the number of genes it contains. Edges represent the degree of similarity and edge thickness is proportional to the similarity between modules. If two communities do not share genes, they are not connected by an edge.

After getting a general context of the enriched functional categories in each network, we narrowed our focus to immune system- and inflammation-related communities. We manually selected those communities based on the criteria that they must contain at least one process related to inflammation or immune response (**Supplementary File 1**). The comparison of the enrichment profiles of communities between different networks show groups of communities with shared enrichment classes. Some of the observed functions are shared between all networks, including the normal tissue network. These groups can be identified as clusters of similar enrichment patterns in the heatmap (**Figures 3**, **4**; **Supplementary Figure 2**).

Given the numerous coincidences in enriched categories between phenotypes, we wanted to know whether similar enrichment patterns between distinct networks were the product of them having equivalent communities formed by similar sets of genes connected in a similar way. We constructed a meta-network with nodes from immune-enriched communities in all networks and defined connections between them based on their structural similarity.

The comparison by gene composition shows a pattern similar to enriched categories. Communities from distinct phenotypes form interconnected groups, some of which have more than 50% of shared genes and correspond to the core of the functional groups In all cases, similarity is smaller than one, which means no module has an identical module in any other network.

The community structure similarity, in which we compare the degree in which genes in similar communities are connected in the same way, confirms the observed patterns, but with overall smaller Jaccard index values, compared to those of gene composition. In this comparison, some of the communities that share genes do not have any equivalent connections, which reflects differences in gene associations between networks at a finer structural level (30).

Here on, we refer to these groups of communities with similar gene composition, connectivity structure, and functional enrichment as *similarity clusters*.

### 3.1 Similarity clusters

Gene compositions and enrichment patterns of each similarity cluster refer to a variety of immunological functions for innate and adaptive immunity responses. Enrichment patterns are also reasonably congruent with the regulation at the gene expression level of an integrated immune response where complementary immune system processes appear, associated to distinct modules (37, 38). These functions include the following: the recruitment of immune system cells represented by genes for chemokines, extracellular matrix components, cell adhesion molecules, and receptors; the capture, processing, and presentation of antigens represented by genes for membrane receptors, immunoproteasome components, and MHC class I and II antigen-presenting molecules (39–41); and immune system effector functions at the cellular and humoral level including genes for cytotoxic enzymes and proteins of the complement system (42–44). Here, we present a summary for each similarity cluster.

Similarity cluster 1 consists of communities: Normal 97 (18 genes), LumA 98 (32 genes), LumB 111 (26 genes), Her2 107 (30 genes), and Basal 102 (28 genes). This cluster contains communities from the four subtypes and normal tissue. The communities in this cluster have a high gene composition similarity between breast cancer subtypes, with Jaccard similarity values between 0.7 and 0.82, and comparatively lower similarity between the normal tissue and the cancer subtypes with values between 0.34 and 0.41. The structural similarity of the communities shows a similar pattern with the highest similarity values between the four breast cancer subtypes and the lowest values between normal tissue and the subtypes.

At the differential expression level, 24 genes are shared by all subtypes, 15 of which are over-expressed in all of them, 4 have no differential expression to normal, and 5 are over-expressed in three out of four of the subtypes (**Supplementary Figure 3**). This cluster contains genes coding for proteins of the histone family (45, 46). The enrichment classes are for chromatin organization and regulation, as well as innate immunity (47). This community is associated with immune response because of the role of histones in antimicrobial responses, like in neutrophil DNA nets (48, 49). All the enrichment categories for this cluster are in association with histone function. None of the communities in this cluster have exclusive enrichment classes.

Similarity cluster 2 is composed of communities: Normal 26 (46 genes), LumA 83 (40 genes), LumB 93 (43 genes), Her2 88 (37 genes), and Basal 104 (25 genes). This cluster contains communities from the four breast cancer subtypes and normal tissue. Gene composition similarity between its communities ranges from 0.52 to 0.33 and structural similarity goes from 0.42 to 0.19. The largest similarity values are found between the communities of the breast cancer subtypes, whereas the lowest values are between the normal tissue module and the breast cancer subtype communities. This cluster contains genes for proteins associated with antiviral response (50) including intracellular pattern recognition receptors (51), interferon receptors, and molecules induced by interferon signaling (52, 53). Enrichment classes include processes associated to antiviral response mediated by interferons.

Similarity cluster 3 includes the following communities: LumA 111 (24 genes), LumB 130 (17 genes), Her2 127 (15 genes) and Basal 137 (11 genes). Gene composition similarity ranges from 0.64 to 0.44 and is reduced in the structural similarity to a range between 0.48 and 0.2. A few genes and enriched categories are shared with Community Normal 392, but it does not share any interactions with LumA 111 and Her2 127 communities. This cluster presents genes for MHC class I molecules (54), as well as TAP1 and TAP2 molecules (55), together with PSMB8 and PSMB9 members of the immunoproteasome, which help in the process of antigen cross-presentation (56, 57). Enrichment classes shared by this cluster share the theme of antigen processing and presentation and the antigen-presenting side of cytotoxic T-cell action (54).

In turn, similarity cluster 4 includes the following communities: Normal 86 (16 genes), LumA 114 (21 genes), LumB 116 (20 genes), Her2 93 (35 genes), and Basal 110 (21 genes). In this cluster, the lowest similarity values by gene composition in the range of 0.27 to 0.33 are from community Her2 93, which is the largest of the group, and the largest values in the range of 0.68 to 0.71 are from the Normal 86 community, which is the smallest. This cluster contains genes for MHC class II molecules, the CIITA gene that controls the MHC class II expression (58), and the CD74 gene involved in MHC class II maturation (59, 60). Enriched processes include inflammation, antigen presentation, and T-cell activation. This cluster appears to represent the antigen-presenting side of the adaptive immune response. Community Her2 93 shares genes and functions in the fourth and seventh similarity clusters (10).

Interestingly, similarity cluster 5, which includes communities Normal 58, LumA 75, LumB 69, Her2 77, and Basal 78, comprises genes for cytokine receptors, components of the T-cell receptor, and the T-cell signaling pathway (61, 62). It also contains genes for the CD8 co-receptor and enzymes of the cytotoxic effector function (Granzymes A, B, H and PRF1) (63, 64). IFNG gene is also part of the communities in the cluster, except for the Normal tissue network.

Similarity cluster 6 includes the following communities: Normal 56 and LumA 122. It includes mostly genes for extracellular matrix components, many of them members of the collagen family. Enrichment categories refer mostly to extracellular matrix organization and the process Regulation of immune response (GO:0050776) appears, perhaps as an artifact of the annotation due to the interaction between immune cells and the extracellular matrix necessary for recruitment.

We can notice also that similarity cluster 7 is less defined with multiple correspondences between networks and an overall lower similitude as shown by Jaccard index calculations. We included the following communities: Normal 21, LumA 80, LumA 125, LumB 70, Her2 93, Her2 111, and Basal 49. Genes in this cluster include numerous cytokines and cytokine receptors (61). Complement system components, like C1QA, C1QB, C1QC, and C4, occur as well as complement receptors C3AR1 and C5AR1. Also present are immunoglobulin receptors FCER1G and FCGR2B and co-receptor CD4. Of these genes, only C1QA, C1QB, C1QC, and C3AR1 are in communities from all five networks (65). Because of the heterogeneity in gene composition, this cluster presents numerous enrichment classes that are not shared between communities or shared by only two or three of them.

Similarity cluster 8 includes the following communities: Normal 10, LumA 84, LumB 120, and Basal 156. This cluster has low levels of similitude between communities and the enrichment categories and genes are not shared across all communities. Enrichment classes in this group include cell signaling molecules, particularly chemokines like CCL2, CCL3, and CCL4, and components of the MAPK pathway including DUSP1, FOSB, JUN, and JUNB (66–68).

Finally, similarity cluster 9 includes the following communities: Normal 61, LumA 112, LumB 119, Her2 104, and Basal 96. Enrichment classes for this cluster share CD79A, SLAMF7, and TNFRSF17 genes in the process Adaptive immune response (GO:0002250). These molecules play a role in cell signaling in lymphocytes (10).

In summary, it is notable that Cluster 4 exhibits the highest degree of gene composition similarity between normal and its subtypes. On the other hand, Her2 shows lower similarity and shares genes with cluster 7 as well. Cluster 1 displays less similarity with tumors, but it exhibits similar immunologic functions within itself. In contrast, Cluster 3 does not exhibit enriched immunologic functions in normal tissue.

### 3.2 Non-shared enrichment classes

Through the comparison of statistically enriched GO processes between networks, we can identify a number of GO terms that are statistically significant in one network, but not significant in any of the other networks.

Basal subtype GO exclusive categories include processes related to immune cell recruitment and activation, mostly through cytokines in the CCL family: CCL2, CCL3, CCL4, CCL19, and CCL21. Other exclusive processes suggest functions of lymphocytes through molecules like FOXP3 and EOMES, which are associated with lymphocyte homeostasis, or BTK, which is part of the B-cell receptor signaling pathway (69, 70). Other processes not directly associated to the immune response but with chromatin structure are represented in module 102, which contains predominantly histone genes.

Her2 subtype GO exclusive categories include processes related to immune cell recruitment and activation, including antigen processing by immunoproteasome, leukocyte migration signals by S100 family genes, and transcriptional control and antiviral response by TRIM family members (71, 72).

Luminal A subtype GO exclusive categories are predominantly related to tissue remodeling and wound healing, angiogenesis, and cell migration. Molecules represented include many components of the extracellular matrix like members of the collagen family COL3A1 and COL1A2 and other ECM components like DCN and VCAN (73). Here, we also find significant enrichment of processes related to DNA modification represented by molecules of the APOBEC family (74, 75).

Luminal B subtype GO exclusive categories include processes related to T-cell response through molecules like the CD4 co-receptor and signal transduction components like FOS, JUN, and JAK3 (76, 77). Other processes associated with immune system signaling or hematopoiesis are represented by HSPA family members HSPA1A and HSPA1B. There are also a number of molecules associated with protein folding and cellular response to heat represented by members of the HSP family (78).

Normal tissue GO exclusive processes are predominantly associated with cell cycle control. These include molecules involved with DNA replication as well as components of the mitotic control and mitotic effector machinery.

### 3.3 Differential expression in immune process-enriched communities

We compared average gene expression levels in the four breast cancer subtypes against the normal phenotype and mapped them to each one of the selected communities in the breast cancer subtypes. The modules show various patterns of differential expression, although many genes show expression levels not significantly different than normal tissue and are the majority in some of the communities.

**Figure 5:**
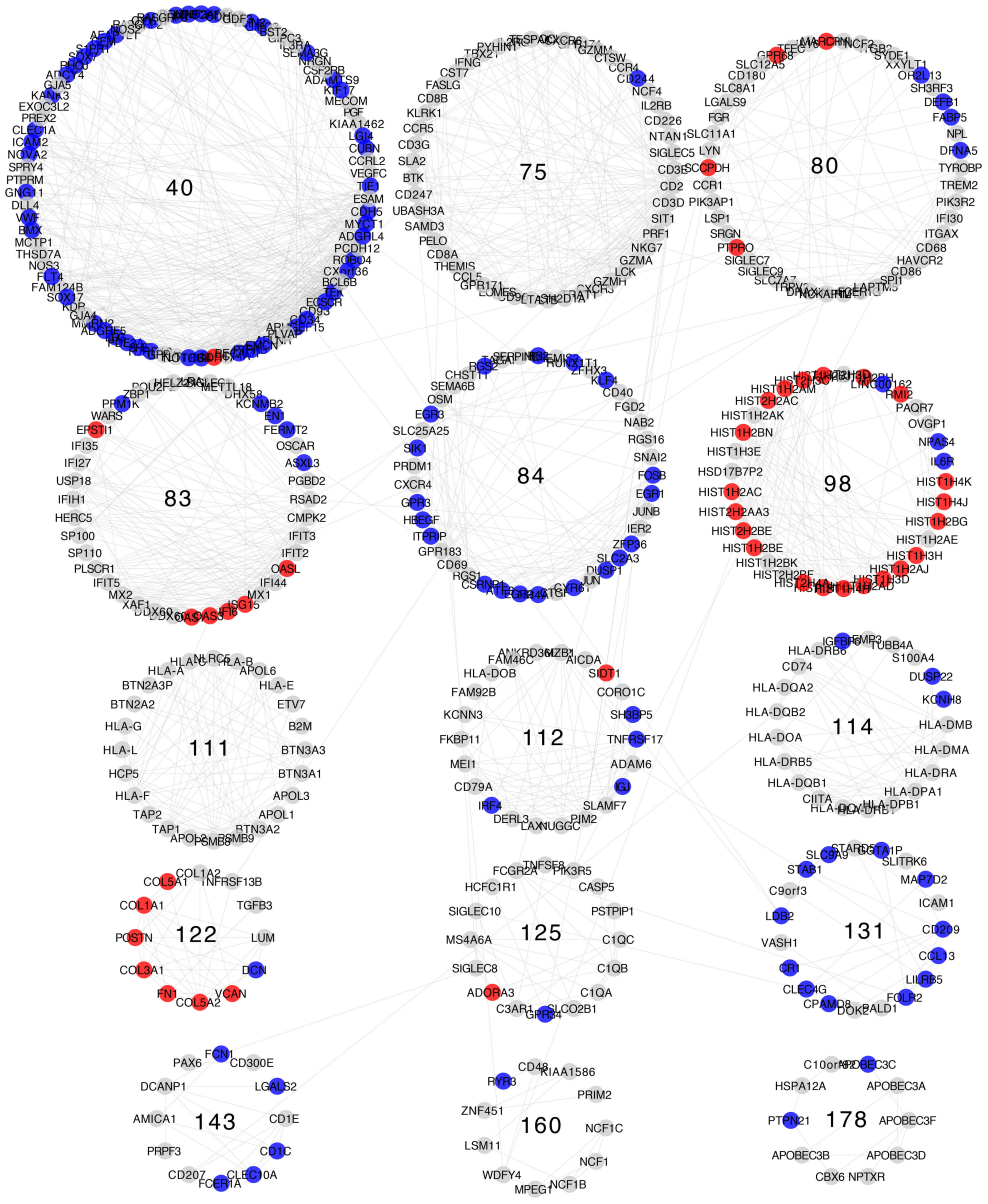
Differential gene expression with respect to normal breast tissue in the communities enriched in inflammation and immune response GO processes from the Luminal A subtype network. Differential expression is similar along genes in the same community, although most communities have genes with many distinct differential expression patterns with respect to normal tissue.

**Figure 6:**
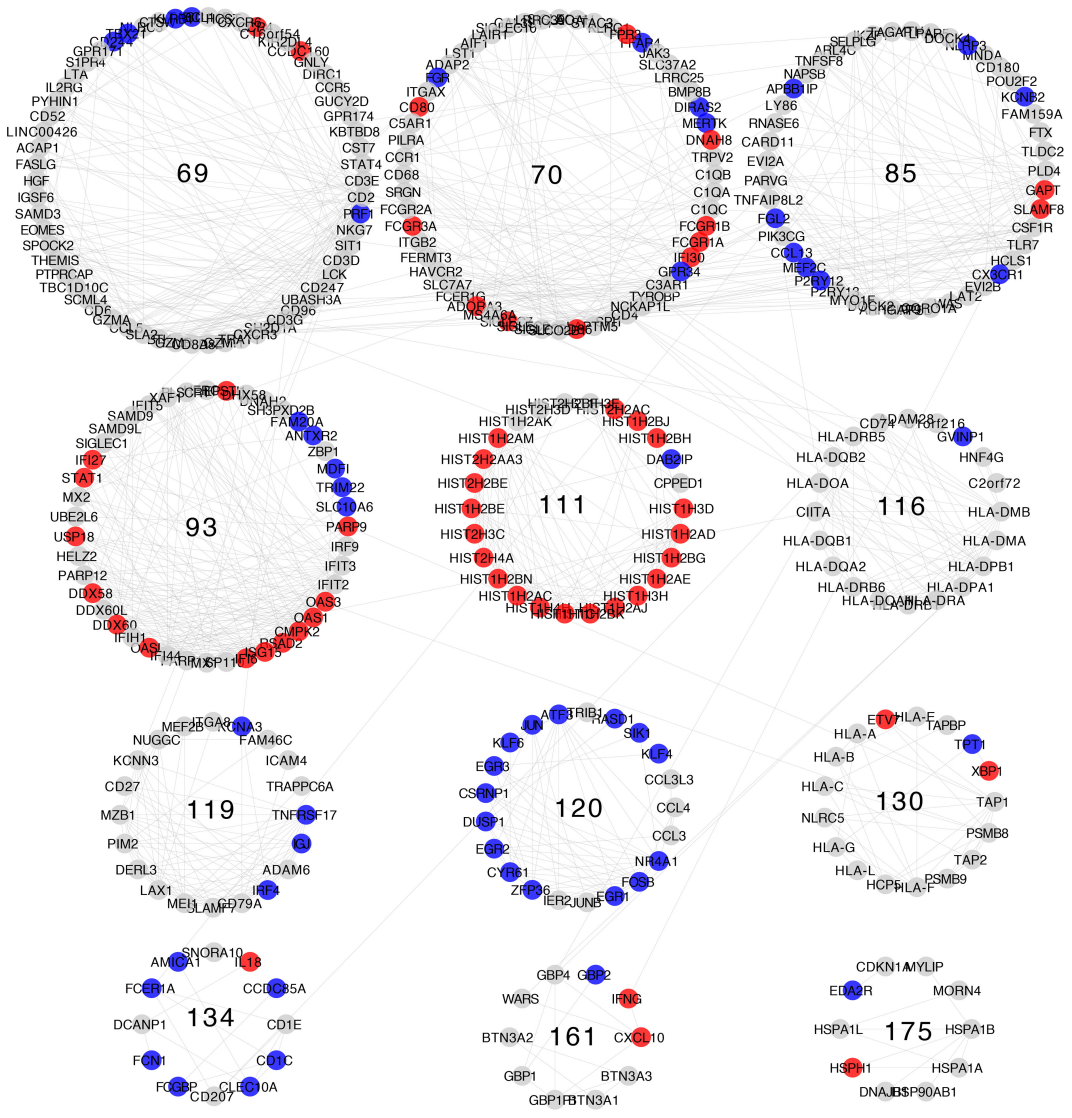
Differential gene expression with respect to normal breast tissue in the communities enriched in inflammation and immune response GO processes from the Luminal B subtype network. Differential expression is similar along genes in the same community, although most communities have genes with many distinct differential expression patterns with respect to normal tissue.

**Figure 7:**
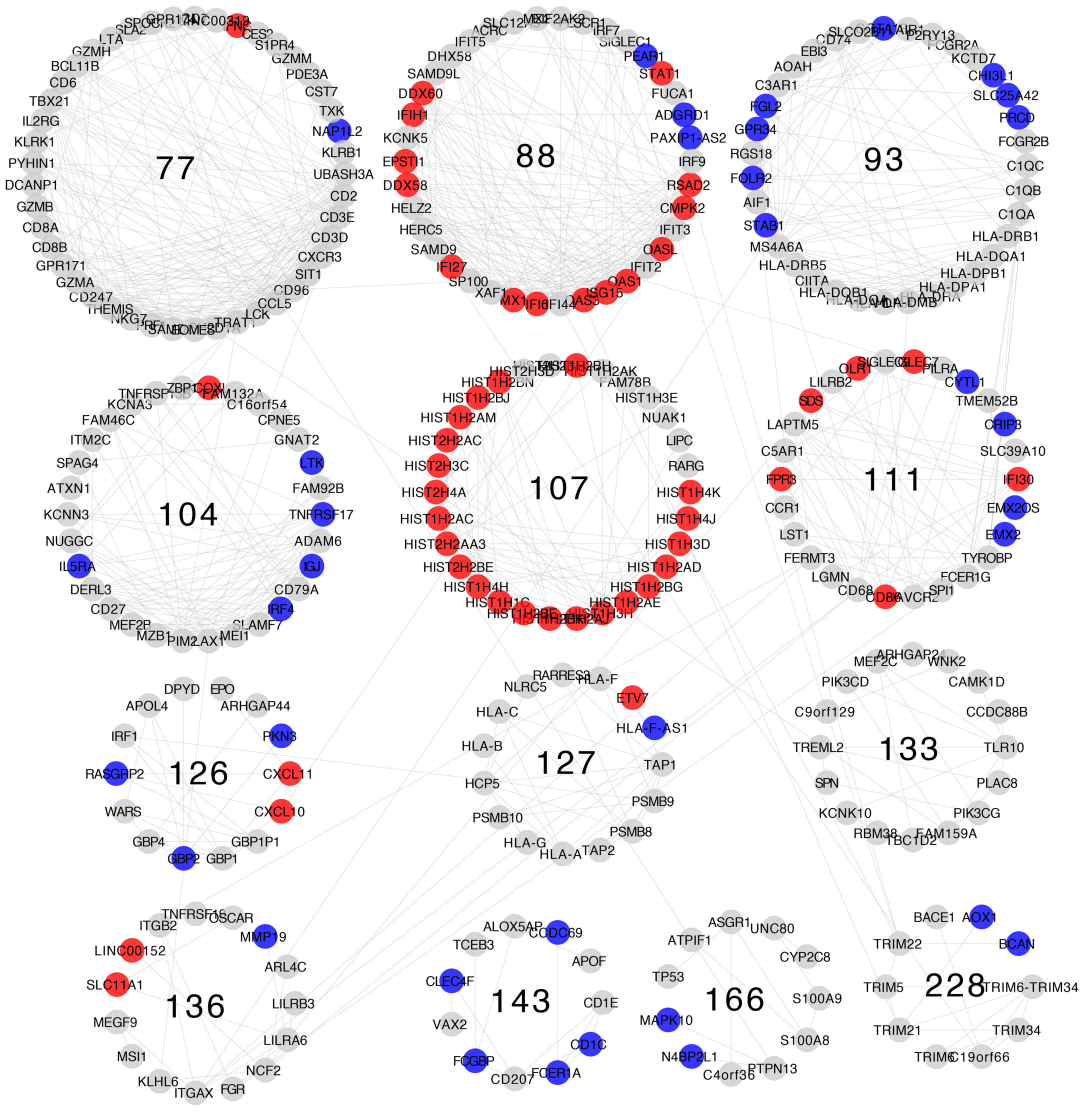
Differential gene expression with respect to normal breast tissue in the communities enriched in inflammation and immune response GO processes from the Her2-enriched subtype network. Differential expression is similar along genes in the same community, although most communities have genes with many distinct differential expression patterns with respect to normal tissue.

**Figure 8:**
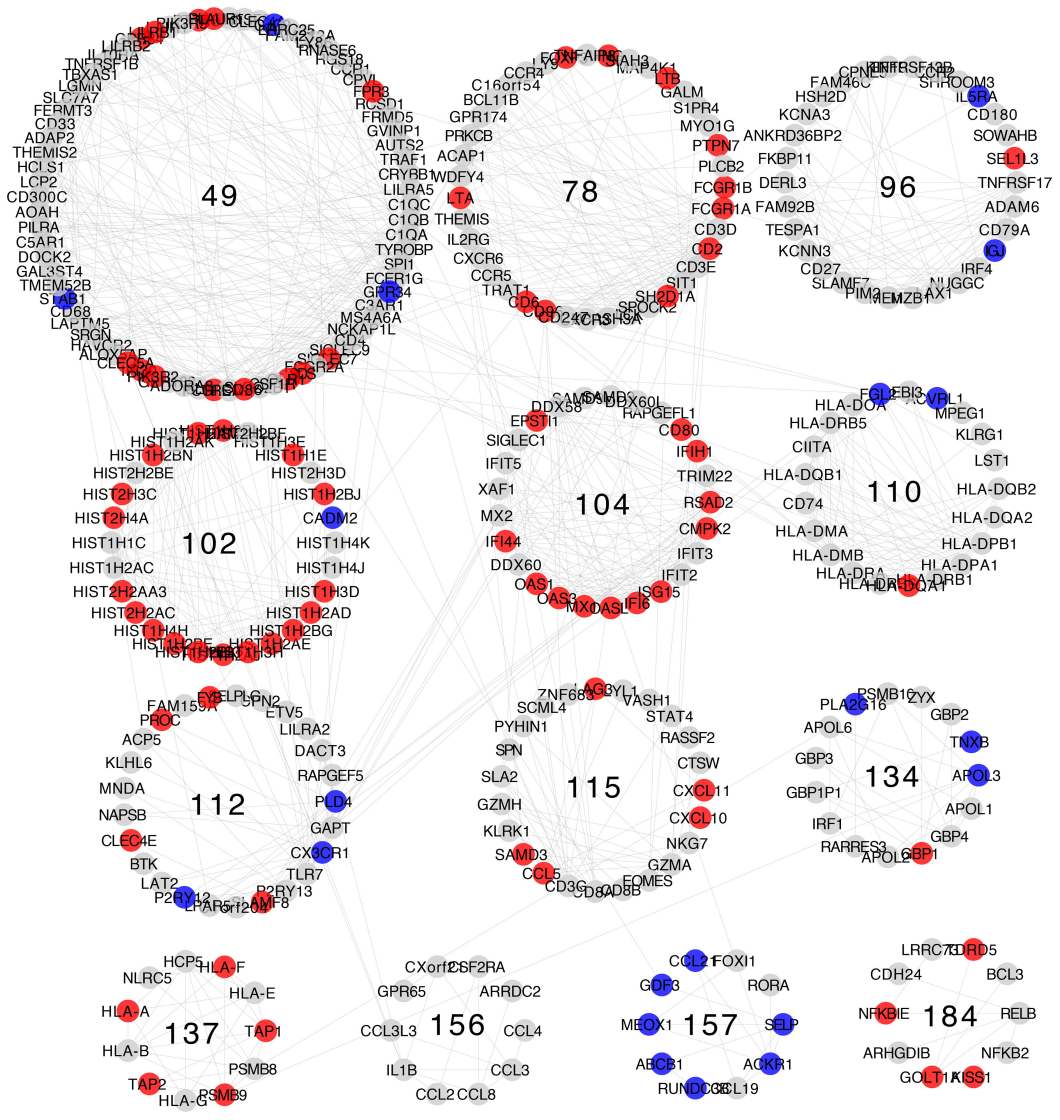
Differential gene expression with respect to normal breast tissue in the communities enriched in inflammation and immune response GO processes from the Basal subtype network. Differential expression is similar along genes in the same community, although most communities have genes with many distinct differential expression patterns with respect to normal tissue.

Differential expression patterns are not completely uniform within each community. Modules show distinct differential expression patterns. In some communities, we can even find examples of over-expressed, under-expressed, and non-differentially expressed genes (**Figures 5**–**8**).

Differential expression patterns tend to be roughly similar between communities belonging to the same similarity cluster. The most evident of which is similarity cluster 1. Modules in this cluster contain mostly over-expressed genes of the histone family. This is the only cluster with both consistent differential expression level and high gene composition similarity across all four breast cancer subtypes.

### 3.4 Differences and commonalities between similarity clusters

**Figure 9:**
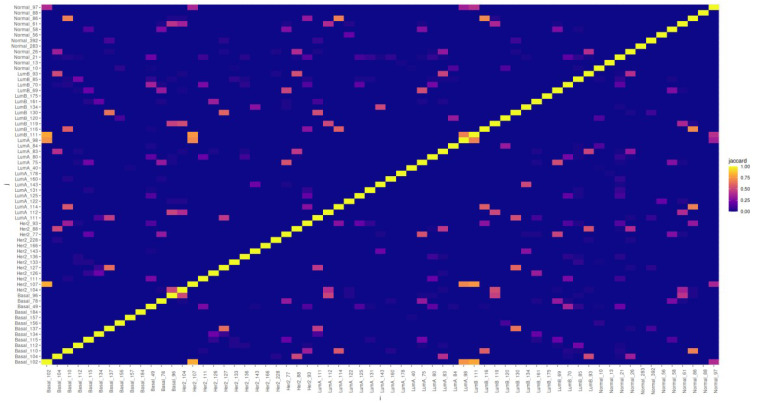
Heatmap matrix presenting Jaccard indices showing the relative intersection of the gene compositions for all similarity clusters discussed. It can be seen that there are a few clusters with relatively high intersections, but most of these have characteristic gene compositions.

The gene compositions of the similarity clusters display some distinctive patterns. We can see in **Figure 9** that most of the clusters are different, sharing none or just a few genes (see also **Supplementary Tables 2**, **3**). However, a few clusters (yellow and orange pixels) may have significantly large intersections, with Jaccard indices up to 0.8125 in the case of the Basal 102 and Her2 107 clusters.

Other clusters showing significant overlap are among the Basal ones: Basal 102 with Lum A 98 (Jaccard index ≃ 0.743), Basal 110 with Normal 96 (Jaccard index ≃ 0.682), and Basal 137 with Lum B 130 (Jaccard index ≃ 0.647). For Her 2 107, the aforementioned similarities were with Basal 102, also with Lum A 98, and Her 2 127 with Basal 137 (Jaccard index ≃ 0.625). For Lum A clusters: Lum A 111 significantly overlaps with Lum B 130 (Jaccard index ≃ 0.519) and Lum A 114 significantly overlaps with Lum B 116 (Jaccard index ≃ 0.577). For Lum B, Lum B 11 significantly overlaps with Her 2 107 (Jaccard index = 0.75).

We can notice (even by a glimpse at **Figure 9**) that in the case of the normal tissue, most clusters have a very small overlap with those of the cancer subtypes, with the notable exception of the intersection of cluster Normal 86 with Basal 110 and Lum A 114 (both with Jaccard index ≃ 0.682) and with Lum B 116 (Jaccard index ≃ 0.714).

## 4 Discussion

This study delves into the complex world of transcriptional regulation within breast cancer molecular subtypes and normal breast tissue, shedding light on how the gene networks differ between these categories. One of the significant findings is the variation in gene composition and structural characteristics of the networks across different subtypes and normal breast tissue. Despite starting with the same gene lists, subtle differences in how genes are associated into co-expression groups referred to here as communities, and the internal structure of these communities, contribute to these variations.

To understand the functional significance of these differences, we have narrowed our focus to communities with significant enrichment categories within each network. It was found that each community exhibits distinct biological processes, consistent with previous research. Interestingly, these communities contain a relatively small number of genes, but the number of enriched processes within them can vary significantly. The comprehensive comparison of enriched categories in different networks effectively highlights the functional separation between these communities (35, 79, 80).

It is relevant to highlight that the present study, which is based on Bulk RNASeq experiments, represents a coarse-grained view that although quite useful is expected to be complemented and in many cases superseded by analysis of single-cell and spatial transcriptomic experiments. At the moment, such experiments are becoming more common, but since higher costs and logistic and experimental complexities continue to prevent them from being applied to large sample sets (many individuals and not just many cells), we believe that the broad view presented here is still quite valuable. Along these lines, we did not just consider genes present in immune cells. This is so since we are convinced that the underlying information that we can gather from analyzing the different cell types in the tumor environment would further illuminate our understanding on these matters. For the present study, hence we did not focus *only* on gene expressed in immune cells since it is very likely that those genes will have associated with other genes and pathways, and by being restricted to this information without considering differences in cell populations, we may be biasing our analysis.

### 4.1 Co-expression modules reveal functional clues about inflammation and immunity

Gene co-expression modules are groups of genes that exhibit coordinated expression patterns across multiple samples or conditions. These modules are identified through probabilistic or functional techniques, where the strength of co-expression relationships between genes is considered. In cancer, these modules can provide valuable insights into the underlying biological processes, pathways, and regulatory networks involved in tumorigenesis and progression. For instance, in He et al. (81), they devised a novel framework to identify distinct patterns of gene co-expression networks and inflammation-related modules from genome-scale microarray data following viral infection. Subsequently, these modules were categorized into oncogenic and dysfunctional types. The core of the framework involves the comparative examination of viral infection modules across various disease stages and types. Module preservation during disease progression is assessed based on alterations in network connectivity across different stages. The evaluation of similarities and differences in HBV and HCV involved comparing the overlap of gene compositions and functional annotations in their respective modules.

The identification of co-expression modules allows researchers to uncover key genes that may act in concert to drive cancer development. These modules often comprise genes with related functions, participating in common biological pathways or cellular processes. Dissecting gene co-expression networks in cancer can reveal potential biomarkers, therapeutic targets, and insights into the heterogeneity of tumors.

Additionally, studying the dynamics of gene co-expression modules across different cancer types or stages can contribute to a better understanding of the molecular diversity within the disease. It aids in the classification of tumors into subtypes based on their gene expression profiles, enabling more personalized and targeted treatment strategies. Overall, the exploration of gene co-expression modules plays a crucial role in deciphering the molecular landscape of cancer and holds promise for advancing our knowledge and improving clinical outcomes. Such is the case of the work described in He et al. (81), which was able to provide novel insights into viral hepatocarcinogenesis and disease progression, underscoring the advantages of an integrative and comparative network analysis over existing approaches reliant on differential expression and virus–host interactome-based methodologies.

Regarding the identification of communities related to inflammation and immune responses, the comparison of community enrichment profiles uncovers shared enrichment classes among different networks, even including the normal tissue network (36, 82). These shared functions are organized into clusters that exhibit similar enrichment patterns. Our findings reveal interconnected groups of communities across various subtypes, some with more than 50% of shared genes, forming the core of functional groups. However, it is crucial to note that no community is identical to another in any network.

Taking this exploration a step further, we assessed the structural similarities between communities by comparing how genes in similar modules are connected (29). This analysis confirms the observed patterns but with overall smaller Jaccard index values. In some instances, communities that share genes do not exhibit equivalent connections, indicating differences in gene associations at a finer structural level. These groups of communities, characterized by shared gene composition, connectivity structure, and functional enrichment (83), are termed similarity clusters. The results of this analysis reveal fascinating insights into the transcriptional regulation of genes within different breast cancer molecular subtypes and normal breast tissue. Each similarity cluster presents distinct characteristics, gene composition, and functional enrichment patterns that are of great significance for our understanding of breast cancer biology.

Similarity Cluster 1 encompasses modules from all breast cancer subtypes and normal tissue. Notably, gene composition similarity is highest between breast cancer subtypes, while normal tissue exhibits lower similarity with the cancer subtypes. The cluster predominantly features genes coding for histone proteins and is associated with immune response due to the role of histones in antimicrobial responses.

In Similarity Cluster 2, communities from all breast cancer subtypes and normal tissue are grouped together. Gene composition similarity is more pronounced between the breast cancer subtype communities, whereas lower similarity values exist between normal tissue and cancer subtypes. This cluster is enriched with genes associated with antiviral responses mediated by interferons.

For Similarity Cluster 3, communities from LumA, LumB, Her2, and Basal subtypes are part of this cluster. Gene composition similarity varies within this cluster, with a few shared genes and enriched categories from Normal 392. This cluster predominantly contains genes related to MHC class I molecules and antigen processing and presentation.

Communities from LumA, LumB, Her2, Basal, and Normal are represented in Similarity Cluster 4. Gene composition similarity varies, with the smallest community (Normal 86) displaying the highest similarity values. This cluster features genes for MHC class II molecules, the CIITA gene, and CD74, enriched in inflammation, antigen presentation, and T-cell activation processes. This result (relatively high similarity between normal tissue and PAM50 subtypes in MHC II molecules) appears paradoxical given that it is known that here is a low level of immune-related surveillance in normal tissue. At the moment, we do not have a definite explanation to this fact, but perhaps this will be better understood in the future by examining, for instance, single-cell and spatial transcriptomics data. One possible explanation is that some of the molecules involved in cancer-related immune surveillance may play additional roles in normal cells. Indeed, of the four main stages of immune surveillance (antigen recognition, immune checkpoint sensing, cytotoxic activity, and memory response), the first and the last (antigen recognition and memory response) may be active even in normal cells, for instance, for pathogen surveillance.

Similarity Cluster 5 comprises genes for cytokine receptors, components of the T-cell receptor, and the T-cell signaling pathway, along with genes related to cytotoxic effector functions common to all subtypes. This cluster is notable for its association with genes promoting immune response.

Similarity Cluster 6 includes communities from Normal and LumA subtypes, primarily characterized by genes related to extracellular matrix components. Enrichment categories focus on extracellular matrix organization and potential immune responses, likely due to interactions between immune cells and the extracellular matrix.

In contrast, Similarity Cluster 7 exhibits comparatively lower similarity values, with communities from Normal, LumA, LumB, Her2, and Basal subtypes. It contains genes for cytokines, cytokine receptors, complement system components, immunoglobulin receptors, and co-receptors. The variety in gene composition results in numerous enrichment classes that may not be shared across all communities.

A similar trend is seen in Similarity Cluster 8, which spans communities from Normal, LumA, LumB, and Basal subtypes; this cluster displays low similarity levels between modules. Enrichment categories include cell signaling molecules and chemokines like CCL2, CCL3, and CCL4, along with components of the MAPK pathway.

Communities from Normal, LumA, LumB, Her2, and Basal subtypes make up Similarity Cluster 9. It features enrichment classes shared by genes like CD79A, SLAMF7, and TNFRSF17 in the context of adaptive immune response.

In summary, these findings highlight the unique gene composition and enrichment patterns within each similarity cluster, offering a comprehensive view of the complex immunological functions associated with breast cancer molecular subtypes and normal breast tissue. Notably, Cluster 4 exhibits the highest gene composition similarity between normal tissue and its subtypes, whereas Her2 displays lower similarity and shares features with Cluster 7. Cluster 1, on the other hand, showcases dissimilarity with tumors but retains shared immunologic functions within its communities. In contrast, Cluster 3 does not exhibit enriched immunologic functions in normal tissue. These results deepen our understanding of the molecular mechanisms at play in breast cancer subtypes, shedding light on their unique characteristics and potential implications for diagnosis and treatment.

We can notice the subtle differences in gene composition and network structure, as well as the importance of shared functional enrichments, which can be interpreted as a global context of the phenotype and providing a foundation for further understanding the regulation of inflammation and immune responses in these phenotypes.

It is interesting to note that, contrary to what one would expect, communities in the network do not correspond to groups of uniformly differentially expressed genes. Complementary to this, genes with similar differential expression tendencies are scattered across a number of communities in the network. We believe this is evidence for the notion that differential expression is not a characteristic that uniquely determines the formation of co-expressed groups. This also opens the question of what other factors may contribute in the formation of such co-expression patterns, especially if we consider the occurrence of distinct co-expressed but non differentially expressed genes, a subtlety that is lost when screening only differentially expressed genes. We can argue that differences in behavior may arise also from differences in the co-expression context of genes, which, in turn, could lead to changes in molecular dynamics and behavior contributing to the expression of a particular phenotype.

This is a broad-level comparison and, as such, is not optimal in the identification of individual genetic actors, but rather to discern general differences in expression patterns between phenotypes. Gene products often have pleiotropic effects, and it seems reasonable they be regulated in many different contexts. This could be one of the causes of the observed multiple connections in our co-expression networks, including those between modules.

We would like to highlight a recurrent observation in genome-wide co-expression enrichment patterns. This is the presence of numerous, highly statistically significant instances of enrichment of immune system categories. In the context of immune response, each cell can be seen as the result of a particular gene-expression program taking effect in the context of the organism. In cancer particularly, the presence of mutations at various levels is recognized as a recurrent and important cause that drives changes in gene expression patterns and the phenotype. In the context of the organism, however, these transformed cells interact with other non-transformed cells affecting their behavior. This is frequently proposed in the form of cancer hijacking normal biological mechanisms to favor its own development. We believe that the recurrence of gene co-expression patterns may be the result of this appropriation of mechanisms by tumors from otherwise genetically normal immune and adjacent normal cells. This may also be one factor causing the observed similarity clusters where we find numerous genes whose expression is restricted to specific cell lineages, specially immune cell lineages.

### 4.2 Further clues from multi-omics

The integration of multi-omics approaches, including DNA methylation assays (84), copy number variants (CNVs) (85, 86), miRNA expression profiling (87–89), transcription factor binding site analysis (90), and proteomics (91), can further improve our understanding of gene regulation in breast cancer tumors (84, 92). These comprehensive techniques offer unprecedented insights into the intricate molecular mechanisms underlying tumorigenesis and progression. Specifically, they illuminate how alterations in DNA methylation patterns, exacerbated DNA copy number variations, dysregulated miRNA expression, aberrant transcription factor binding, and dysregulated protein expression contribute to the development and behavior of breast cancer. Importantly, these multi-omics approaches also shed light on the potential role of gene regulation in modulating immune responses within the tumor microenvironment. By uncovering key molecular players involved in immune evasion or activation, multi-omics analyses provide valuable insights for the development of novel immunotherapeutic strategies aimed at harnessing the immune system to combat breast cancer.

As previously noted, previous work form our group and others has analyzed such multi-omic effects on gene regulation in breast cancer subtypes at a large scale in several of these scenarios (92). However, a multi-omic study centered around immune responses in breast cancer subtypes has been missing. As an initial approach to the problem, we harmonized and integrated multi-omic TCGA data for the breast cancer subtypes studied in this project (see code and further relevant information in the following online repository: https://github.com/josemaz/omicsBRCA/) and applied a fairly general statistical framework to analyze it on an integrated manner (84), namely, a sparse generalized canonical correlation analysis (SGCCA) (see associated code in https://github.com/CSB-IG/SGCCA). **Supplementary Table 4** presents some significant results.

Interestingly, and in contrast with analyses based purely on gene composition, which show a relatively low number of significant similarities between the similarity clusters, but more closely in line with functional enrichment analyses, it was found that a number of multi-omic regulatory interactions exists for these clusters and there is a non-trivial overlap between those. Our studies highlight the roles that multitargeted transcription factors, miRNA regulators, and epigenomic phenomena (mostly in the form of hypomethylated regions) play in *consolidating* biological functions in the similarity clusters.

For brevity, we must only comment on some of these relationships; however, the full set of SGCCA statistically significant multi-omic calculated associations can be found in **Supplementary Table 4**. Note that the microRNA hsa-mir-146a-5p is a highly significant common multi-omic regulator of the Basal 104, Her2 88, LumA 83, Lum B 93, and Normal 26 similarity clusters (technical note: *p*-values have been *capped* in **Supplementary Table 4**, so that any *p*-value less than 1*E*−16 appears displayed as 0 [*zero*]). In addition, hsa-mir-152–3p is a common regulator of the Her2 127 and Lum A 111 clusters.

Perhaps the most consistent finding (though not surprising at all) is that transcription factors of the IRF family are master regulators of many similarity clusters, most of them in cancer subtypes (with the exception of the Normal 26 cluster). In terms of epigenomic phenomena, we found that there is significant hypomethylation in promoter regions of the MTF-1 gene associated with the expression patterns in the Basal 102 and Lum B 116 clusters.

However, more comprehensive analyses need to be performed in order to unveil the full potential of multi-omics to reveal the extent of immune-related transcriptional regulatory processes in breast tumor subtypes. It is very likely that these future studies may involve single-cell multi-omic descriptions.

### 4.3 On the role of copy number variants in gene co-expression patterns

Since breast cancer molecular subtypes have been shown to be enriched for specific cytobands and chromosome arm amplifications or deletions, it is relevant to consider the effects that CNVs may have in co-expression patterns. The role of CNVs and other structural variations of the genome has been well-documented for decades now (though methods—both experimental and computational—to assess the extent and boundaries of structural genome variation have been significantly improved in recent times). It is expected that these will also affect gene co-expression patterns. Our research group has been carefully analyzing CNVs in the context of breast cancer from the same sample sets considered in this study (the TCGA collaboration has also DNA sequencing and CNV calling data for their breast cancer projects) recently. We have observed for certain genomic regions and/or breast cancer subtypes that the influence of CNVs on gene co-expression is less significant than we have expected (85, 86, 93).

These studies are on themselves challenging long-term projects before strong conclusions can be reached. However, we can argue that the effect of CNVs in breast cancer co-expression may likely explain just a fraction of the associated variance.

### 4.4 Potential strategies for experimental validation

The objective of computational systems biology approaches, such as the one outlined here, is often to propose a promising set of hypotheses for subsequent testing and validation through targeted experiments in more controlled environments. Our aim is to generate plausible findings that highlight semi-mechanistic processes, facilitating experimental validation to advance our comprehension of the phenomena and assess the reliability of our computational methods. In the past, through systems biology-oriented analyses, some of these findings were found by our group and others (84, 91, 94–98). However, the ultimate benchmark in natural sciences remains experimental validation, reproducibility, and, to some extent, generalizability. Several potential experimental methodologies may include proteomic, phospho-proteomic, and functional activity measurements.

While these finely targeted measurements are intricate to execute, there are emerging studies. For instance, Debets et al. (99) have recently used a deep coverage phosphoproteomic strategy to identify immune-related signatures of treatment resistance in HER2+ breast cancer. A similar approach was used (with lower depth coverage) to characterize TBK regulation in innate immune signaling in triple-negative breast cancer (100). Broader analyses have been performed to analyze signaling -*kinome* activity in luminal breast tumors (101). Phosphorylation and immune signaling were also studied to characterize the effect of mutation profiles (102), immune biomarkers (103), and potential for pharmacological therapy (104).

Toll-like receptor activity patterns have been globally studied considering immune biomarkers in breast tumors in a multicentric proteome study (105) with a clear clinical goal, but comprehensive basic studies have also been performed in relation to the activity of cancer-associated fibroblasts’ presence and absence (106), immune escape by GATA3 destabilization (107), and follicular helper cells as promoters of effective adaptive immunity (108).

### 4.5 Limitations of this study

Breast cancer is a highly heterogeneous disease. Among the sources of variability in phenotype presentation, therapeutic response, and overall outcomes, some are related to ancestry and the genetic makeup of the underlying populations. The TCGA–Genomic Data Commons database is one of the largest, most comprehensive and curated repositories of omic data for cancer, including breast tumors. Although it includes diverse populations, most samples belong to US residents and are thus enriched in Caucasian ancestry. In spite of this and other design considerations, it spans a lot of variability that can be indeed stratified for its well-curated metadata, both clinically and regarding other determinants of health. With this in mind, we used TCGA as our primary dataset and indeed we were able to adapt this to our own designs by further classifying and prioritizing the particular samples we used in our study. That was the reason to further reclassify samples with more stringent algorithms. Although the issue of generalizability of results just described is quite relevant, we believe that these analyses will provide enough insight to serve as a starting point for deeper, broader research.

We consider relevant to recognize the origin of the data and the limitations of the variables measured. Our data come from fine needle aspiration (FNA) samples taken from living tissue. These samples contain a number of different cells that are representative of the cells present within the tissue and processed in bulk. Because of it, measured mRNA abundances reflect small regions of the tissue of origin and not specific cell types. This is a technical limitation we hope gets resolved with more recent single-cell sequencing technologies.

Moreover, we have measured abundances of RNA species assigned to known genes, which we assume as a proxy for protein abundance. This, however, does not directly account for mutations, splicing variants, and post-translational modifications, although the active modulation of cell behavior through these processes is hinted in the structure and gene composition of the networks.

## 5 Conclusions

In summary, our study allowed us to shed some light to further understand the complex and distinctive transcriptional networks within various breast cancer molecular subtypes and normal breast tissue. By conducting an in-depth analysis, we have identified nine similarity clusters of gene communities whose transcriptional signatures (some of them similar among themselves and even to normal healthy tumor-adjacent tissue, in terms of co-expression patterns) may contribute to characterize the immunological response patterns (as reflected in gene co-expression activity) shared, as well as those inherent to each subtype.

The presence of both innate and adaptive immune responses may reflect a coordinated immunological defense mechanism against the disease. These immunological signatures not only may deepen our comprehension of the similarities and differences among subtypes, but also are able to potentially advance our understanding of the relevant functional features towards the development of personalized diagnostic and therapeutic strategies.

Furthermore, our network-based approach provides a valuable framework for dissecting the complexities involved in breast cancer-associated immune responses, paving the way to uncover their underlying biology. As research continues, we hope that these insights may be gradually translated into tangible clinical benefits, ultimately improving patient outcomes and transforming the current approach to cancer management.

## Data availability statement

The original contributions presented in the study are included in the article/**Supplementary Material**. Further inquiries can be directed to the corresponding author.

## Ethics statement

The studies involving humans were approved by NCI/NHGRI TCGA Data Access Committee -for further information please communicate to tcgadac@mail.nih.gov. The studies were conducted in accordance with the local legislation and institutional requirements. Written informed consent for participation was not required from the participants or the participants’ legal guardians/next of kin in accordance with the national legislation and institutional requirements.

## Author contributions

TV-C: Data curation, Methodology, Software, Validation, Visualization, Writing – original draft, Writing – review & editing. JZ-F: Formal Analysis, Investigation, Writing – review & editing, Data curation. EH-L: Conceptualization, Formal Analysis, Funding acquisition, Investigation, Project administration, Supervision, Writing – original draft, Writing – review & editing.

## Supplementary material

Supplementary File 1 Excel spreadsheet with enrichment results for communities with immune-related categories

Supplementary Figure 1 Heatmap comparing all enriched categories from all networks. Communities with immune -related enrichments are labelled in red.

Supplementary Figure 2 Heatmap comparing enrichments in all immune-related modules between all networks.

Supplementary Figure 3 Chart comparing DE status of genes from cluster 1 present in all cancer phenotypes.

Scripts used to perform these analyses are publicly available at the following repositories:

https://github.com/tadeito/breastcancerimmunenetworks.

https://github.com/josemaz/omicsBRCA.

https://github.com/CSB-IG/SGCCA.

## References

[1] DeNardo DG Coussens LM. Inflammation and breast cancer. balancing immune response: crosstalk between adaptive and innate immune cells during breast cancer progression. Breast Cancer Res. (2007) 9:1–10. doi: 10.1186/bcr1746 PMC2206719 17705880

[2] Jézéquel P Loussouarn D Guérin-Charbonnel C Campion L Vanier A Gouraud W. Gene-expression molecular subtyping of triple-negative breast cancer tumours: importance of immune response. Breast Cancer Res. (2015) 17:1–16. doi: 10.1186/s13058-015-0550-y 25887482 PMC4389408

[3] Akimoto M Ishii H Nakajima Y Iwasaki H Tan M Abe R. Assessment of host immune response in breast cancer patients. Cancer detection Prev. (1986) 9:311–7. 3488806

[4] Luen S Virassamy B Savas P Salgado R Loi S. The genomic landscape of breast cancer and its interaction with host immunity. Breast. (2016) 29:241–50. doi: 10.1016/j.breast.2016.07.015 27481651

[5] Savas P Salgado R Denkert C Sotiriou C Darcy PK Smyth MJ. Clinical relevance of host immunity in breast cancer: from tils to the clinic. Nat Rev Clin Oncol. (2016) 13:228–41. doi: 10.1038/nrclinonc.2015.215 26667975

[6] Wang M Zhang C Song Y Wang Z Wang Y Luo F. Mechanism of immune evasion in breast cancer. OncoTargets Ther. (2017) 10:1561–73. doi: 10.2147/OTT.S126424 PMC5359138 28352189

[7] Bates JP Derakhshandeh R Jones L webb TJ. Mechanisms of immune evasion in breast cancer. BMC Cancer. (2018) 18:1–14. doi: 10.1186/s12885-018-4441-3 29751789 PMC5948714

[8] Wu SZ Roden DL Wang C Holliday H Harvey K Cazet AS. Stromal cell diversity associated with immune evasion in human triple-negative breast cancer. EMBO J. (2020) 39:e104063. doi: 10.15252/embj.2019104063 32790115 PMC7527929

[9] Jiang X Shapiro DJ. The immune system and inflammation in breast cancer. Mol Cell Endocrinol. (2014) 382:673–82. doi: 10.1016/j.mce.2013.06.003 PMC4919022 23791814

[10] Gatti-Mays ME Balko JM Gameiro SR Bear HD Prabhakaran S Fukui J. If we build it they will come: targeting the immune response to breast cancer. NPJ Breast Cancer. (2019) 5:37. doi: 10.1038/s41523-019-0133-7 31700993 PMC6820540

[11] McDonald K-A Kawaguchi T Qi Q Peng X Asaoka M Young J. Tumor heterogeneity correlates with less immune response and worse survival in breast cancer patients. Ann Surg Oncol. (2019) 26:2191–9. doi: 10.1245/s10434-019-07338-3 PMC6545241 30963401

[12] Lan H-R Du W-L Liu Y Mao C-S Jin K-T Yang X. Role of immune regulatory cells in breast cancer: foe or friend? Int Immunopharmacol. (2021) 96:107627. doi: 10.1016/j.intimp.2021.107627 33862552

[13] Perou CM Sørlie T Eisen MB Van De Rijn M Jeffrey SS Rees CA. Molecular portraits of human breast tumours. nature. (2000) 406:747–52. doi: 10.1038/35021093 10963602

[14] Sørlie T Perou CM Tibshirani R Aas T Geisler S Johnsen H. Gene expression patterns of breast carcinomas distinguish tumor subclasses with clinical implications. Proc Natl Acad Sci. (2001) 98:10869–74. doi: 10.1073/pnas.191367098 PMC58566 11553815

[15] Chia SK Bramwell VH Tu D Shepherd LE Jiang S Vickery T. A 50-gene intrinsic subtype classifier for prognosis and prediction of benefit from adjuvant tamoxifen. Clin Cancer Res. (2012) 18:4465–72. doi: 10.1158/1078-0432.CCR-12-0286 PMC3743663 22711706

[16] Yang Y Han L Yuan Y Li J Hei N Liang H. Gene co-expression network analysis reveals common system-level properties of prognostic genes across cancer types. Nat Commun. (2014) 5:3231. doi: 10.1038/ncomms4231 24488081 PMC3951205

[17] Tang J Kong D Cui Q Wang K Zhang D Gong Y. Prognostic genes of breast cancer identified by gene co-expression network analysis. Front Oncol. (2018) 8:374. doi: 10.3389/fonc.2018.00374 30254986 PMC6141856

[18] Ben-Dor A Bruhn L Friedman N Nachman I Schummer M Yakhini Z. (2000). Tissue classification with gene expression profiles, in: Proceedings of the fourth annual international conference on Computational molecular biology, RECOMB conferences, New York, USA. pp. 54–64. 10.1089/106652700750050943 11108479

[19] Namy O Rousset J-P Napthine S Brierley I. Reprogrammed genetic decoding in cellular gene expression. Mol Cell. (2004) 13:157–68. doi: 10.1016/s1097-2765(04)00031-0 14759362

[20] Niehrs C Luke B. Regulatory r-loops as facilitators of gene expression and genome stability. Nat Rev Mol Cell Biol. (2020) 21:167–78. doi: 10.1038/s41580-019-0206-3 PMC7116639 32005969

[21] Hsu H-M Chu C-M Chang Y-J Yu J-C Chen C-T Jian C-E. Six novel immunoglobulin genes as biomarkers for better prognosis in triple-negative breast cancer by gene co-expression network analysis. Sci Rep. (2019) 9:4484. doi: 10.1038/s41598-019-40826-w 30872752 PMC6418134

[22] García-Cortés D de Anda-Jáuregui G Fresno C Hernandez-Lemus E Espinal-Enriquez J. Gene co-expression is distance-dependent in breast cancer. Front. Oncol. (2020) 10:1232. doi: 10.3389/fonc.2020.01232 PMC7396632 32850369

[23] González-Espinoza A Zamora-Fuentes J Hernández-Lemus E Espinal-Enríquez J. Gene co-expression in breast cancer: A matter of distance. Front Oncol. (2021) 11:726493. doi: 10.3389/fonc.2021.726493 34868919 PMC8636045

[24] Tomczak K Czerwińska P Wiznerowicz M. The cancer genome atlas (tcga): an immeasurable source of knowledge. Contemp Oncol. (2015) 19:A68. doi: 10.5114/wo.2014.47136 PMC4322527 25691825

[25] Espinal-Enriquez J Fresno C Anda-Jáuregui G Hernández-Lemus E. Rna-seq based genome-wide analysis reveals loss of inter-chromosomal regulation in breast cancer. Sci Rep. (2017) 7:1–19. doi: 10.1038/s41598-017-01314-1 28496157 PMC5431987

[26] Fresno C González GA Merino GA Flesia AG Podhajcer OL Llera AS. A novel non-parametric method for uncertainty evaluation of correlation-based molecular signatures: its application on pam50 algorithm. Bioinformatics. (2017) 33:693–700. doi: 10.1093/bioinformatics/btw704 28062443

[27] Hernández-Lemus E Siqueiros-Garcıa J. Information theoretical methods for complex network structure reconstruction, complex adap. Syst Mod. (2013) 1:693–700. doi: 10.1186/2194-3206-1-8

[28] Margolin AA Nemenman I Basso K Wiggins C Stolovitzky G Dalla Favera R. Aracne: an algorithm for the reconstruction of gene regulatory networks in a mammalian cellular context. In: BMC bioinformatics, vol. 7. New York, USA: Springer (2006). p. S7. 10.1186/1471-2105-7-S1-S7 PMC1810318 16723010

[29] de Anda-Jáuregui G Alcalá-Corona SA Espinal-Enríquez J Hernández-Lemus E. Functional and transcriptional connectivity of communities in breast cancer co-expression networks. Appl Network Sci. (2019) 4:1–13. doi: 10.1007/s41109-019-0129-0

[30] Alcalá-Corona SA Sandoval-Motta S Espinal-Enriquez J Hernandez-Lemus E. Modularity in biological networks. Front Genet. (2021) 12:701331. doi: 10.3389/fgene.2021.701331 34594357 PMC8477004

[31] Rosvall M Bergstrom CT. Maps of information flow reveal community structure in complex networks. arXiv preprint physics.soc-ph/0707.0609. (2007). 10.1073/pnas.0611034104 PMC1855072 17452639

[32] Rosvall M Bergstrom CT. Maps of random walks on complex networks reveal community structure. Proc Natl Acad Sci. (2008) 105:1118–23. doi: 10.1073/pnas.0706851105 PMC2234100 18216267

[33] Consortium GO. Gene ontology consortium: going forward. Nucleic Acids Res. (2015) 43:D1049–56. doi: 10.1093/nar/gku1179 PMC4383973 25428369

[34] Ritchie ME Phipson B Wu D Hu Y Law CW Shi W. limma powers differential expression analyses for rna-sequencing and microarray studies. Nucleic Acids Res. (2015) 43:e47–7. doi: 10.1093/nar/gkv007 PMC4402510 25605792

[35] de Anda-Jáuregui G Velázquez-Caldelas TE Espinal-Enríquez J Hernández-Lemus E. Transcriptional network architecture of breast cancer molecular subtypes. Front Physiol. (2016) 7:568. doi: 10.3389/fphys.2016.00568 27920729 PMC5118907

[36] Alcalá-Corona SA de Anda-Jáuregui G Espinal-Enríquez J Hernández-Lemus E. Network modularity in breast cancer molecular subtypes. Front Physiol. (2017) 8:915. doi: 10.3389/fphys.2017.00915 29204123 PMC5699328

[37] Korkaya H Liu S Wicha MS. Breast cancer stem cells, cytokine networks, and the tumor microenvironment. J Clin Invest. (2011) 121:3804–9. doi: 10.1172/JCI57099 PMC3223613 21965337

[38] Ascierto ML Kmieciak M Idowu MO Manjili R Zhao Y Grimes M. A signature of immune function genes associated with recurrence-free survival in breast cancer patients. Breast Cancer Res Treat. (2012) 131:871–80. 10.1007/s10549-011-1470-x PMC3431022 21479927

[39] Forero A Li Y Chen D Grizzle WE Updike KL Merz ND. Expression of the mhc class ii pathway in triple-negative breast cancer tumor cells is associated with a good prognosis and infiltrating lymphocytes. Cancer Immunol Res. (2016) 4:390–9. doi: 10.1158/2326-6066.CIR-15-0243 PMC4878913 26980599

[40] Park IA Hwang S-H Song IH Heo S-H Kim Y-A Bang WS. Expression of the mhc class ii in triple-negative breast cancer is associated with tumor-infiltrating lymphocytes and interferon signaling. PloS One. (2017) 12:e0182786. doi: 10.1371/journal.pone.0182786 28817603 PMC5560630

[41] Axelrod ML Cook RS Johnson DB Balko JM. Biological consequences of mhc-ii expression by tumor cells in cancer. Clin Cancer Res. (2019) 25:2392–402. doi: 10.1158/1078-0432.CCR-18-3200 PMC6467754 30463850

[42] Hecht F Pessoa CF Gentile LB Rosenthal D Carvalho DP Fortunato RS. The role of oxidative stress on breast cancer development and therapy. Tumor Biol. (2016) 37:4281–91. doi: 10.1007/s13277-016-4873-9 26815507

[43] Afshar-Kharghan V. The role of the complement system in cancer. J Clin Invest. (2017) 127:780–9. doi: 10.1172/JCI90962 PMC5330758 28248200

[44] Reis ES Mastellos DC Ricklin D Mantovani A Lambris JD. Complement in cancer: untangling an intricate relationship. Nat Rev Immunol. (2018) 18:5–18. doi: 10.1038/nri.2017.97 28920587 PMC5816344

[45] Hsieh C-L Ma H-P Su C-M Chang Y-J Hung W-Y Ho Y-S. Alterations in histone deacetylase 8 lead to cell migration and poor prognosis in breast cancer. Life Sci. (2016) 151:7–14. doi: 10.1016/j.lfs.2016.02.092 26926079

[46] Feng J Meng X. Histone modification and histone modification-targeted anti-cancer drugs in breast cancer: Fundamentals and beyond. Front Pharmacol. (2022) 13:946811. doi: 10.3389/fphar.2022.946811 36188615 PMC9522521

[47] Fritz AJ Ghule PN Boyd JR Tye CE Page NA Hong D. Intranuclear and higher-order chromatin organization of the major histone gene cluster in breast cancer. J Cell Physiol. (2018) 233:1278–90. doi: 10.1002/jcp.25996 PMC5705002 28504305

[48] Rivera-Franco MM Leon-Rodriguez E Torres-Ruiz JJ Gómez-Martín D Angles-Cano E de la Luz Sevilla-González M. Neutrophil extracellular traps associate with clinical stages in breast cancer. Pathol Oncol Res. (2020) 26:1781–5. doi: 10.1007/s12253-019-00763-5 31656990

[49] Cristinziano L Modestino L Antonelli A Marone G Simon H-U Varricchi G. Neutrophil extracellular traps in cancer. In Semin Cancer Biol (Elsevier) vol. (2022) 79:91–104. doi: 10.1038/nri.2017.105 34280576

[50] Bhatelia K Singh K Singh R. Tlrs: linking inflammation and breast cancer. Cell signalling. (2014) 26:2350–7. doi: 10.1016/j.cellsig.2014.07.035 25093807

[51] Nabet BY Qiu Y Shabason JE Wu TJ Yoon T Kim BC. Exosome rna unshielding couples stromal activation to pattern recognition receptor signaling in cancer. Cell. (2017) 170:352–66. doi: 10.1016/j.cell.2017.06.031 PMC6611169 28709002

[52] Wu Y Zhao W Liu Y Tan X Li X Zou Q. Function of hnrnpc in breast cancer cells by controlling the dsrna-induced interferon response. EMBO J. (2018) 37:e99017. doi: 10.15252/embj.201899017 30158112 PMC6276880

[53] Sceneay J Goreczny GJ Wilson K Morrow S DeCristo MJ Ubellacker JM. Interferon signaling is diminished with age and is associated with immune checkpoint blockade efficacy in triple-negative breast cancer. Cancer Discovery. (2019) 9:1208–27. doi: 10.1158/2159-8290.CD-18-1454 PMC11167954 31217296

[54] Luo N Nixon MJ Gonzalez-Ericsson PI Sanchez V Opalenik SR Li H. Dna methyltransferase inhibition upregulates mhc-i to potentiate cytotoxic t lymphocyte responses in breast cancer. Nat Commun. (2018) 9:248. doi: 10.1038/s41467-017-02630-w 29339738 PMC5770411

[55] Henle AM Nassar A Puglisi-Knutson D Youssef B Knutson KL. Downregulation of tap1 and tap2 in early stage breast cancer. PloS One. (2017) 12:e0187323. doi: 10.1371/journal.pone.0187323 29091951 PMC5706630

[56] Adwal A Kalita-de Croft P Shakya R Lim M Kalaw E Taege LD. Tradeoff between metabolic i-proteasome addiction and immune evasion in triple-negative breast cancer. Life Sci Alliance. (2020) 3:1–12. doi: 10.26508/lsa.201900562 PMC7240743 32423906

[57] Geoffroy K Araripe Saraiva B Viens M Béland D Bourgeois-Daigneault M-C. Increased expression of the immunoproteasome subunits psmb8 and psmb9 by cancer cells correlate with better outcomes for triple-negative breast cancers. Sci Rep. (2023) 13:2129. doi: 10.1038/s41598-023-28940-2 36746983 PMC9902398

[58] Mostafa AA Codner D Hirasawa K Komatsu Y Young MN Steimle V. Activation of er*α* signaling differentially modulates ifn-*γ* induced hla-class ii expression in breast cancer cells. PloS One. (2014) 9:e87377. doi: 10.1371/journal.pone.0087377 24475282 PMC3903652

[59] Richard V Kindt N Decaestecker C Gabius H-J Laurent G Noel J-C. Involvement of macrophage migration inhibitory factor and its receptor (cd74) in human breast cancer. Oncol Rep. (2014) 32:523–9. doi: 10.3892/or.2014.3272 PMC4091881 24939415

[60] Wang Z-Q Milne K Webb JR Watson PH. Cd74 and intratumoral immune response in breast cancer. Oncotarget. (2017) 8:12664. doi: 10.18632/oncotarget.8610 27058619 PMC5355043

[61] Esquivel-Velázquez M Ostoa-Saloma P Palacios-Arreola MI Nava-Castro KE Castro JI Morales-Montor J. The role of cytokines in breast cancer development and progression. J Interferon Cytokine Res. (2015) 35:1–16. doi: 10.1089/jir.2014.0026 25068787 PMC4291218

[62] Chen W Qin Y Liu S. Cytokines, breast cancer stem cells (bcscs) and chemoresistance. Clin Trans Med. (2018) 7:1–13. doi: 10.1186/s40169-018-0205-6 PMC6119679 30175384

[63] Paydarnia N Khoshtinat Nikkhoi S Fakhravar A Mehdiabdol M Heydarzadeh H Ranjbar S. Synergistic effect of granzyme b-azurin fusion protein on breast cancer cells. Mol Biol Rep. (2019) 46:3129–40. doi: 10.1007/s11033-019-04767-x 30937652

[64] Jin YW Hu P. Tumor-infiltrating cd8 t cells predict clinical breast cancer outcomes in young women. Cancers. (2020) 12:1076. doi: 10.3390/cancers12051076 32357420 PMC7281139

[65] Mangogna A Agostinis C Bonazza D Belmonte B Zacchi P Zito G. Is the complement protein c1q a pro-or anti-tumorigenic factor? bioinformatics analysis involving human carcinomas. Front Immunol. (2019) 10:865. doi: 10.3389/fimmu.2019.00865 31130944 PMC6509152

[66] Boulding T Wu F McCuaig R Dunn J Sutton CR Hardy K. Differential roles for dusp family members in epithelial-to-mesenchymal transition and cancer stem cell regulation in breast cancer. PloS One. (2016) 11:e0148065. doi: 10.1371/journal.pone.0148065 26859151 PMC4747493

[67] Zhang R Li X Liu Z Wang Y Zhang H Xu H. Ezh2 inhibitors-mediated epigenetic reactivation of fosb inhibits triple-negative breast cancer progress. Cancer Cell Int. (2020) 20:1–11. doi: 10.1186/s12935-020-01260-5 32477007 PMC7236314

[68] Rogic A Pant I Grumolato L Fernandez-Rodriguez R Edwards A Das S. High endogenous ccl2 expression promotes the aggressive phenotype of human inflammatory breast cancer. Nat Commun. (2021) 12:6889. doi: 10.1038/s41467-021-27108-8 34824220 PMC8617270

[69] Fiori Lopes L Losi Guembarovski R Guembarovski AL Okuyama Kishima M Campos CZ Oda JMM. Foxp3 transcription factor: a candidate marker for susceptibility and prognosis in triple negative breast cancer. BioMed Res Int. (2014) 2014:1–7. doi: 10.1155/2014/341654 PMC4022106 24877082

[70] Kalaw E Lim M Kutasovic JR Sokolova A Taege L Johnstone K. Metaplastic breast cancers frequently express immune checkpoint markers foxp3 and pd-l1. Br J Cancer. (2020) 123:1665–72. doi: 10.1038/s41416-020-01065-3 PMC7686342 32939056

[71] Barczak W Rozwadowska N Romaniuk A Lipińska N Lisiak N Grodecka-Gazdecka S. Telomere length assessment in leukocytes presents potential diagnostic value in patients with breast cancer. Oncol Lett. (2016) 11:2305–9. doi: 10.3892/ol.2016.4188 PMC4774613 26998167

[72] Bai X Tang J. Trim proteins in breast cancer: Function and mechanism. Biochem Biophys Res Commun. (2023) 640:26–31. doi: 10.1016/j.bbrc.2022.11.103 36495607

[73] Lepucki A Orlińska K Mielczarek-Palacz A Kabut J Olczyk P Komosińska-Vassev K. The role of extracellular matrix proteins in breast cancer. J Clin Med. (2022) 11:1250. doi: 10.3390/jcm11051250 35268340 PMC8911242

[74] Mao Y Lv M Zhang Y Nie G Cui J Wang Y. Apobec3b expression and its prognostic potential in breast cancer. Oncol Lett. (2020) 19:3205–14. doi: 10.3892/ol.2020.11433 PMC7074638 32256817

[75] Vitiello GAF de Sousa Pereira N Amarante MK Banin-Hirata BK Campos CZ de Oliveira KB. Germline apobec3b deletion influences clinicopathological parameters in luminal-a breast cancer: Evidences from a southern Brazilian cohort. J Cancer Res Clin Oncol. (2020) 146:1523–32. doi: 10.1007/s00432-020-03208-8 PMC11804360 32285256

[76] Yuan C-L Jiang X-M Yi Y Jian-Fei E Zhang N-D Luo X. Identification of differentially expressed lncrnas and mrnas in luminal-b breast cancer by rna-sequencing. BMC Cancer. (2019) 19:1–12. doi: 10.1186/s12885-019-6395-5 31795964 PMC6889534

[77] López-Mejía JA Mantilla-Ollarves JC Rocha-Zavaleta L. Modulation of jakstat signaling by lnk: A forgotten oncogenic pathway in hormone receptor-positive breast cancer. Int J Mol Sci. (2023) 24:14777. doi: 10.3390/ijms241914777 37834225 PMC10573125

[78] Klimczak M Biecek P Zylicz A Zylicz M. Heat shock proteins create a signature to predict the clinical outcome in breast cancer. Sci Rep. (2019) 9:7507. doi: 10.1038/s41598-019-43556-1 31101846 PMC6525249

[79] Wolf DM Lenburg ME Yau C Boudreau A van ‘t Veer LJ. Gene co-expression modules as clinically relevant hallmarks of breast cancer diversity. PloS One. (2014) 9:e88309. doi: 10.1371/journal.pone.0088309 24516633 PMC3917875

[80] Thorsson V Gibbs DL Brown SD Wolf D Bortone DS Yang T-HO. The immune landscape of cancer. Immunity. (2018) 48:812–30. 10.1016/j.immuni.2018.03.023 PMC5982584 29628290

[81] He D Liu Z-P Honda M Kaneko S Chen L. Coexpression network analysis in chronic hepatitis b and c hepatic lesions reveals distinct patterns of disease progression to hepatocellular carcinoma. J Mol Cell Biol. (2012) 4:140–52. doi: 10.1093/jmcb/mjs011 22467683

[82] Ozturk K Dow M Carlin DE Bejar R Carter H. The emerging potential for network analysis to inform precision cancer medicine. J Mol Biol. (2018) 430:2875–99. doi: 10.1016/j.jmb.2018.06.016 PMC6097914 29908887

[83] de Anda-Jáuregui G. Guideline for comparing functional enrichment of biological network modular structures. Appl Network Sci. (2019) 4:1–17. doi: 10.1007/s41109-019-0128-1

[84] Ochoa S Hernández-Lemus E. Functional impact of multi-omic interactions in breast cancer subtypes. Front Genet. (2023) 13:1078609. doi: 10.3389/fgene.2022.1078609 36685900 PMC9850112

[85] Hernández-Gómez C Hernández-Lemus E Espinal-Enríquez J. Cnvs in 8q24. 3 do not influence gene co-expression in breast cancer subtypes. Front Genet. (2023) 14:1141011. doi: 10.3389/fgene.2023.1141011 37274786 PMC10236314

[86] Hernández-Gómez C Hernández-Lemus E Espinal-Enríquez J. The role of copy number variants in gene co-expression patterns for luminal b breast tumors. Front Genet. (2022) 13:806607. doi: 10.3389/fgene.2022.806607 35432489 PMC9010943

[87] de Anda-Jáuregui G Espinal-Enríquez J Hernández-Lemus E. Highly connected, non-redundant microrna functional control in breast cancer molecular subtypes. Interface Focus. (2021) 11:20200073. doi: 10.1098/rsfs.2020.0073 34123357 PMC8193465

[88] de Anda-Jáuregui G Espinal-Enríquez J Drago-García D Hernández-Lemus E. Nonredundant, highly connected micrornas control functionality in breast cancer networks. Int J Genomics. (2018) 2018:1–10. doi: 10.1155/2018/9585383 PMC5996465 30003085

[89] Drago-García D Espinal-Enríquez J Hernández-Lemus E. Network analysis of emt and met micro-rna regulation in breast cancer. Sci Rep. (2017) 7:13534. doi: 10.1038/s41598-017-13903-1 29051564 PMC5648819

[90] Trujillo-Ortíz R Espinal-Enríquez J Hernández-Lemus E. The role of transcription factors in the loss of inter-chromosomal co-expression for breast cancer subtypes. Int J Mol Sci. (2023) 24:17564. doi: 10.3390/ijms242417564 38139393 PMC10743684

[91] Ruhle M Espinal-Enríquez J Hernández-Lemus E. The breast cancer protein co-expression landscape. Cancers. (2022) 14:2957. doi: 10.3390/cancers14122957 35740621 PMC9221059

[92] Ochoa S Hernández-Lemus E. Molecular mechanisms of multi-omic regulation in breast cancer. Front Oncol. (2023) 13. doi: 10.3389/fonc.2023.1148861 PMC10411627 37564937

[93] Garcia-Cortes D Hernandez-Lemus E Espinal Enríquez J. Loss of long-range co-expression is a common trait in cancer. bioRxiv. (2022), 2022–10. doi: 10.1101/2022.10.27.513947 PMC13247121 41935098

[94] de Kruijf EM Engels CC van de Water W Bastiaannet E Smit VT van de Velde CJ. Tumor immune subtypes distinguish tumor subclasses with clinical implications in breast cancer patients. Breast Cancer Res Treat. (2013) 142:355–64. doi: 10.1007/s10549-013-2752-2 24197659

[95] Tapia-Carrillo D Tovar H Velazquez-Caldelas TE Hernandez-Lemus E. Master regulators of signaling pathways: an application to the analysis of gene regulation in breast cancer. Front Genet. (2019) 10:474787. doi: 10.3389/fgene.2019.01180 PMC6902642 31850059

[96] Tekpli X Lien T Røssevold AH Nebdal D Borgen E Ohnstad HO. An independent poor-prognosis subtype of breast cancer defined by a distinct tumor immune microenvironment. Nat Commun. (2019) 10:5499. doi: 10.1038/s41467-019-13329-5 31796750 PMC6890706

[97] García-Cortés D Hernández-Lemus E Espinal-Enríquez J. Luminal a breast cancer co-expression network: Structural and functional alterations. Front Genet. (2021) 12:629475. doi: 10.3389/fgene.2021.629475 33959148 PMC8096206

[98] Onkar SS Carleton NM Lucas PC Bruno TC Lee AV Vignali DA. The great immune escape: understanding the divergent immune response in breast cancer subtypes. Cancer Discovery. (2023) 13:23–40. doi: 10.1158/2159-8290.CD-22-0475 36620880 PMC9833841

[99] Debets DO Stecker KE Piskopou A Liefaard MC Wesseling J Sonke GS. Deep (phospho) proteomics profiling of pre-treatment needle biopsies identifies signatures of treatment resistance in her2+ breast cancer. Cell Rep Med. (2023) 4:1–16. doi: 10.1016/j.xcrm.2023.101203 PMC10591042 37794585

[100] Ritter JL Zhu Z Thai TC Mahadevan NR Mertins P Knelson EH. Phosphorylation of rab7 by tbk1/ikk-e regulates innate immune signaling in triple-negative breast cancer. Cancer Res. (2020) 80:44–56. doi: 10.1158/0008-5472.CAN-19-1310 31662325 PMC6942622

[101] Yang G Zuo C Lin Y Zhou X Wen P Zhang C. Comprehensive proteome, phosphoproteome and kinome characterization of luminal a breast cancer. Front Oncol. (2023) 13:1127446. doi: 10.3389/fonc.2023.1127446 37064116 PMC10102592

[102] De Marchi T Lai C-F Simmons GM Goldsbrough I Harrod A Lam T. Proteomic profiling reveals that esr1 mutations enhance cyclin-dependent kinase signaling. Sci Rep. (2024) 14:6873. doi: 10.1038/s41598-024-56412-8 38519482 PMC10959978

[103] Terkelsen T Pernemalm M Gromov P Børresen-Dale A-L Krogh A Haakensen VD. High-throughput proteomics of breast cancer interstitial fluid: identification of tumor subtype-specific serologically relevant biomarkers. Mol Oncol. (2021) 15:429–61. doi: 10.1002/1878-0261.12850 PMC7858121 33176066

[104] Huang K-l Wu Y Primeau T Wang Y-T Gao Y McMichael JF. Regulated phosphosignaling associated with breast cancer subtypes and druggability*[s]. Mol Cell Proteomics. (2019) 18:1630–50. doi: 10.1074/mcp.RA118.001243 PMC6682998 31196969

[105] El Bairi K Haynes HR Blackley E Fineberg S Shear J Turner S. The tale of tils in breast cancer: a report from the international immuno-oncology biomarker working group. NPJ Breast Cancer. (2021) 7:150. doi: 10.1038/s41523-021-00346-1 34853355 PMC8636568

[106] Dou D Ren X Han M Xu X Ge X Gu Y. Cancer-associated fibroblasts-derived exosomes suppress immune cell function in breast cancer via the mir-92/pd-l1 pathway. Front Immunol. (2020) 11:2026. doi: 10.3389/fimmu.2020.02026 33162971 PMC7581790

[107] Zhang M Wang N Song P Fu Y Ren Y Li Z. Lncrna gata3-as1 facilitates tumour progression and immune escape in triple-negative breast cancer through destabilization of gata3 but stabilization of pd-l1. Cell proliferation. (2020) 53:e12855. doi: 10.1111/cpr.12855 32687248 PMC7507373

[108] Noël G Fontsa ML Garaud S De Silva P De Wind A Van den Eynden GG. Functional th1-oriented t follicular helper cells that infiltrate human breast cancer promote effective adaptive immunity. J Clin Invest. (2021) 131:1–17. doi: 10.1172/JCI139905 PMC8483751 34411002

