# Supplementary figures and images for: Coordinated inflammation and immune response transcriptional regulation in breast cancer molecular subtypes
Source: Front Immunol. 2024 Jun 25;15:1357726. doi: 10.3389/fimmu.2024.1357726 (PMC11231215; doi:10.3389/fimmu.2024.1357726)

Comparison All Enriched Modules In All Networks

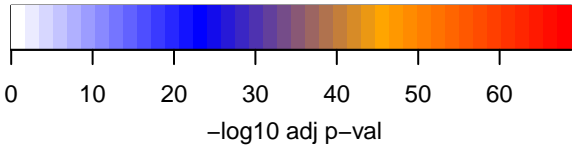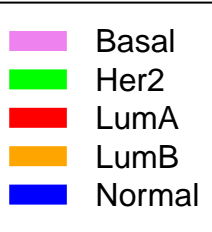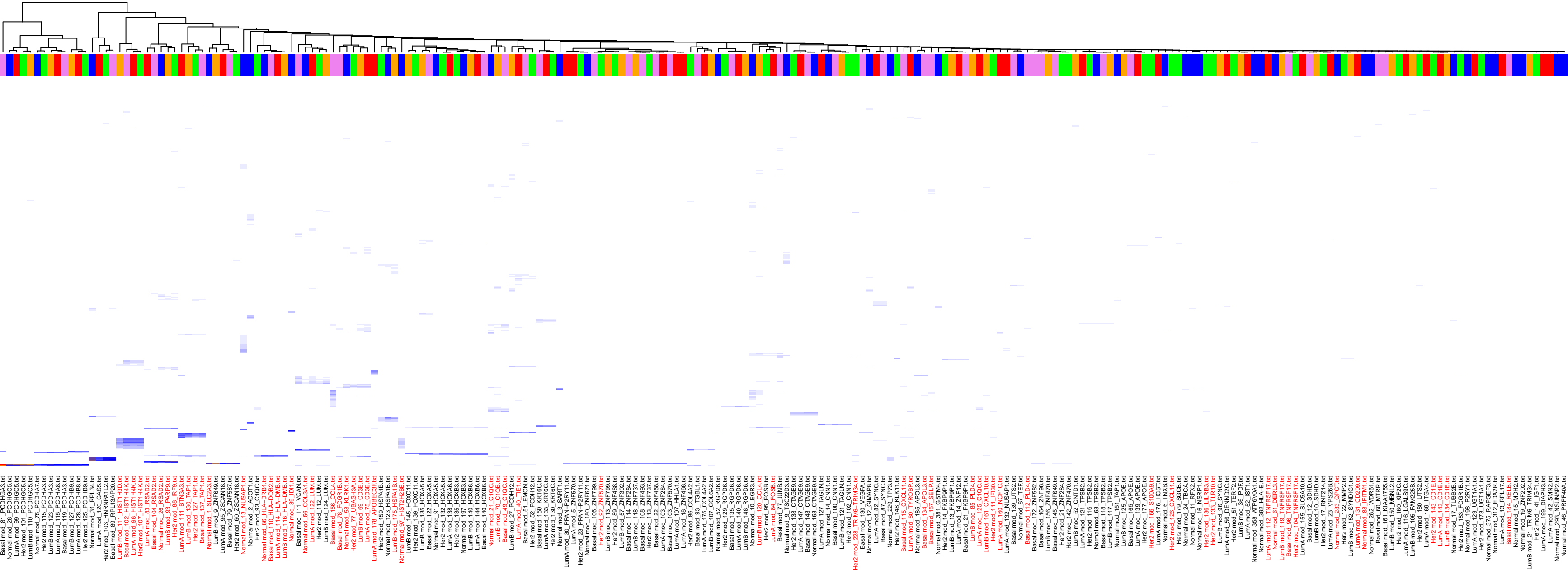

GO Terms

Module

Supplement: Supplementary Figure 1 — Heatmap comparing all enriched categories from all networks. Communities with immune -related enrichments are labelled in red. [file DataSheet_1.pdf]

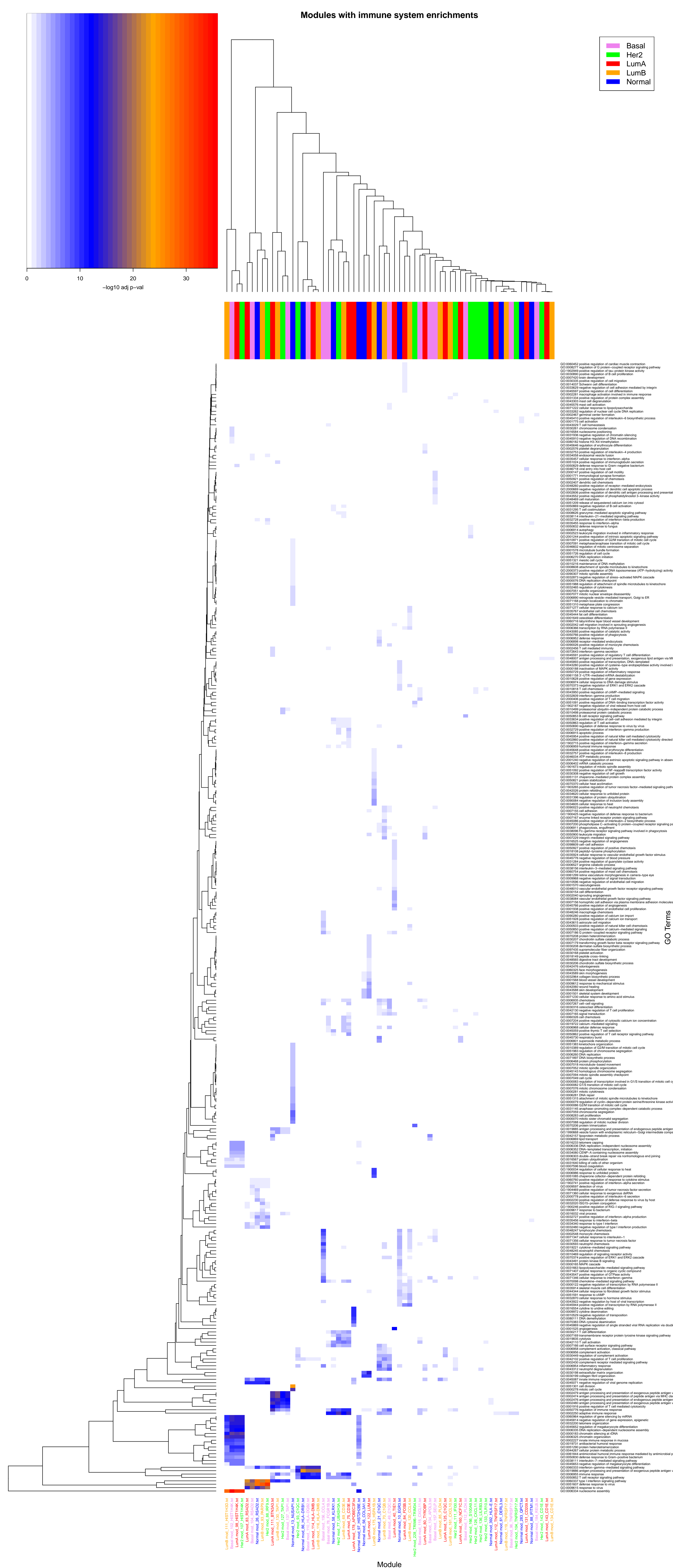

Supplement: Supplementary Figure 2 — Heatmap comparing enrichments in all immune-related modules between all networks. [file DataSheet_2.pdf]

Cluster 1

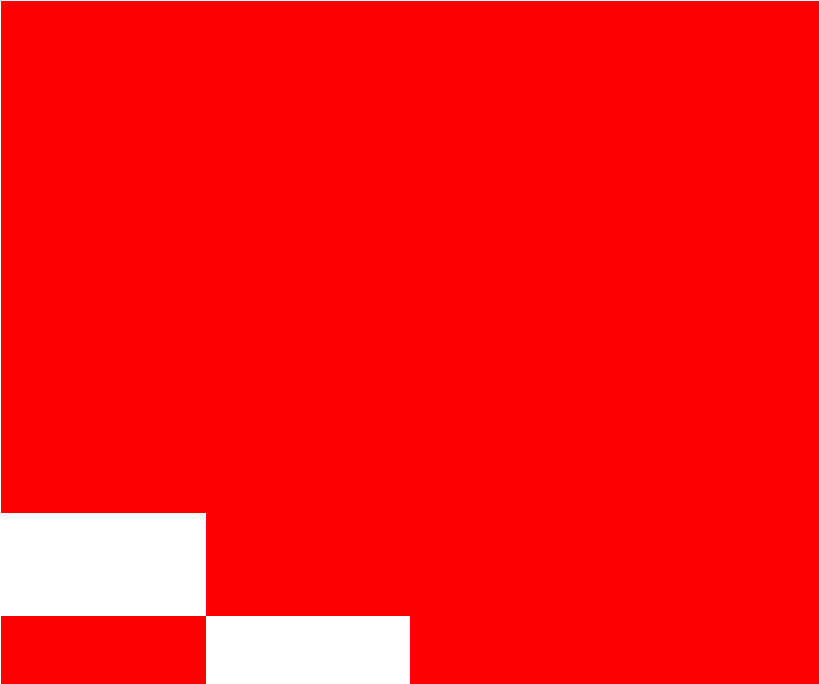

Basal

LumA

LumB

Her2

Supplement: Supplementary Figure 3 — Chart comparing DE status of genes from cluster 1 present in all cancer phenotypes. [file DataSheet_3.pdf]
